# Anti-atherosclerotic effects and molecular targets of salvianolic acids from *Salvia miltiorrhiza* Bunge

**DOI:** 10.3389/fphar.2025.1574086

**Published:** 2025-05-21

**Authors:** Qingzhi Liang, Xiaoqin Liu, Xin Xu, Zhengtao Chen, Ting Luo, Yi Su, Hongyan Xie, Hong Gao, Chunguang Xie

**Affiliations:** ^1^ Department of Endocrinology, Hospital of Chengdu University of Traditional Chinese Medicine, Chengdu, Sichuan, China; ^2^ Department of Emergency, Hospital of Chengdu University of Traditional Chinese Medicine, Chengdu, Sichuan, China; ^3^ Affiliated Hospital of Jiangxi University of Chinese Medicine, Nanchang, Jiangxi, China; ^4^ TCM Regulating Metabolic Diseases Key Laboratory of Sichuan Province, Hospital of Chengdu University of Traditional Chinese Medicine, Chengdu, Sichuan, China

**Keywords:** *Salvia miltiorrhiza* Bunge, salvianolic acid A, salvianolic acid B, danshensu, atherosclerosis

## Abstract

*Salvia miltiorrhiza* Bunge [Lamiaceae; Salviae miltiorrhizae radix et rhizoma] is a traditional Chinese medication used extensively as a therapeutic agent against atherosclerosis (AS) because of its substantial cardiovascular protective properties as well as ability to regulate the signaling cascades and molecular targets involved in AS. Preclinical research has shown that the hydrophilic metabolites of *S. miltiorrhiza* Bunge represented by danshensu (DSS), salvianolic acid A (SAA), and salvianolic acid B (SAB) can reduce endothelial dysfunction, inhibit smooth muscle cell migration and proliferation, block platelet aggregation, have antithrombotic properties, and modulate vascular tone. Furthermore, studies have shown that salvianolic acid is clinically beneficial, while some evidence also supports its safety and effectiveness in diseases linked to AS. The present study is a review of the anti-atherosclerotic pharmacological activities, pharmacokinetic characteristics, drug interactions, and safety evaluations of salvianolic acid over the last 20 years. Herein, we focus on the cellular targets linked to AS; clarify the molecular mechanisms of the anti-atherosclerotic activities of DSS, SAA, and SAB; and discuss the future needs and priorities in light of the limitations of the existing studies. This review is intended to establish the groundwork and offer a thorough perspective for deeper investigations of the studies, clinical uses, and product development efforts of salvianolic acid as a natural modulator of AS.

## 1 Introduction

**FIGURE 1:**
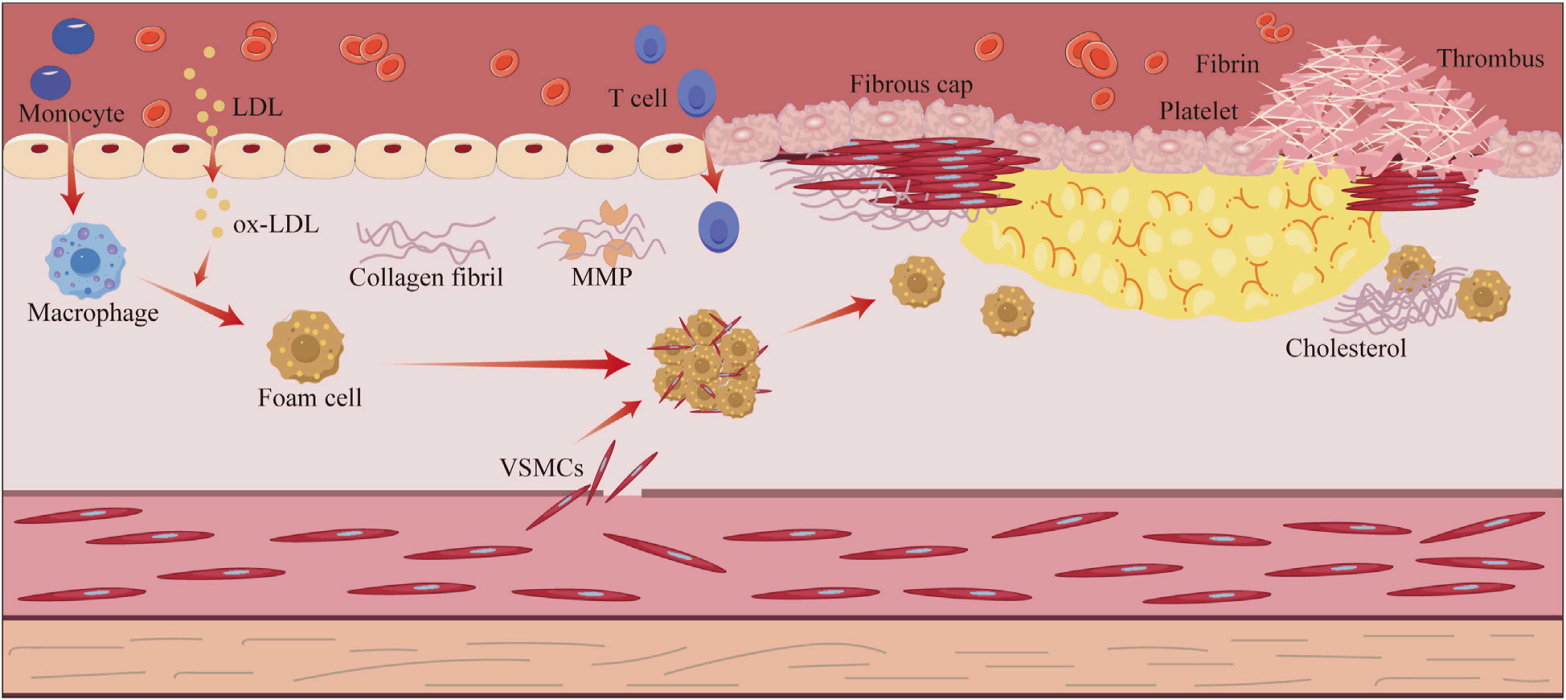
Schematic overview of the pathological processes related to atherosclerosis. Atherosclerosis begins with endothelial damage triggered by risk factors, such as hypertension, hyperglycemia, and hyperlipidemia. The increased permeability of the damaged endothelium encourages infiltration of low-density lipoprotein (LDL) into the endothelium, which is converted to oxidized LDL. Monocytes migrate to the endothelium and differentiate into macrophages, which phagocytose the oxidized LDL and transform into foam cells, forming lipid streaks. Subsequently, smooth muscle cells migrate to the intima and secrete collagen, which wraps around the lipid core to form a fibrous plaque. The gradual increase in plaque size leads to narrowing of the arterial lumen; when inflammation disrupts the fibrous cap, the plaque ruptures and exposes the lipid core, ultimately triggering platelet aggregation and thrombus formation.

Atherosclerosis (AS) constitutes a multifaceted pathological process characterized by chronic non-specific inflammation within the arterial walls (Wolf and Ley, 2019). The core pathogenesis of AS entails a complex interplay of lipid metabolism dysregulation, vascular endothelial dysfunction, inflammatory cell infiltration, and subsequent foam cell formation, ultimately culminating in atherosclerotic plaque development and vascular remodeling (Figure 1) (Mauersberger et al., 2022; Orecchioni et al., 2022; Wang et al., 2015a; Xu et al., 2021; Zheng et al., 2017). Epidemiological evidence has consistently demonstrated that AS-related cardiovascular diseases, including coronary heart disease, myocardial infarction, and stroke, remain the primary causes of mortality globally (Souilhol et al., 2018). Notably, the incidence of these AS-associated diseases continues to escalate in developing countries, posing significant public health challenges (Cainzos-Achirica et al., 2020; Poller et al., 2020). Current anti-AS therapies like statins, proprotein convertase subtilisin/kexin type 9 (PCSK-9) inhibitors, and antiplatelet agents has certain limitations: statins are known to cause myotoxicity (1.9%–30% incidence) and hepatotoxicity; aspirin increases the risk of gastrointestinal bleeding (9.4%); clopidogrel shows resistance due to CYP2C19 polymorphism; PCSK-9 inhibitors are costly, and their interventional procedures have high restenosis rates (34.8%–73.1%) and expenses (Abd and Jacobson, 2011; Ariesen et al., 2006; Kashani et al., 2006; Montastruc, 2023; Pradhan et al., 2024; Turner and Pirmohamed, 2019; Ward et al., 2019; Wu et al., 2017). These challenges highlight the urgent need for safer multitarget anti-AS drugs.

**FIGURE 2:**
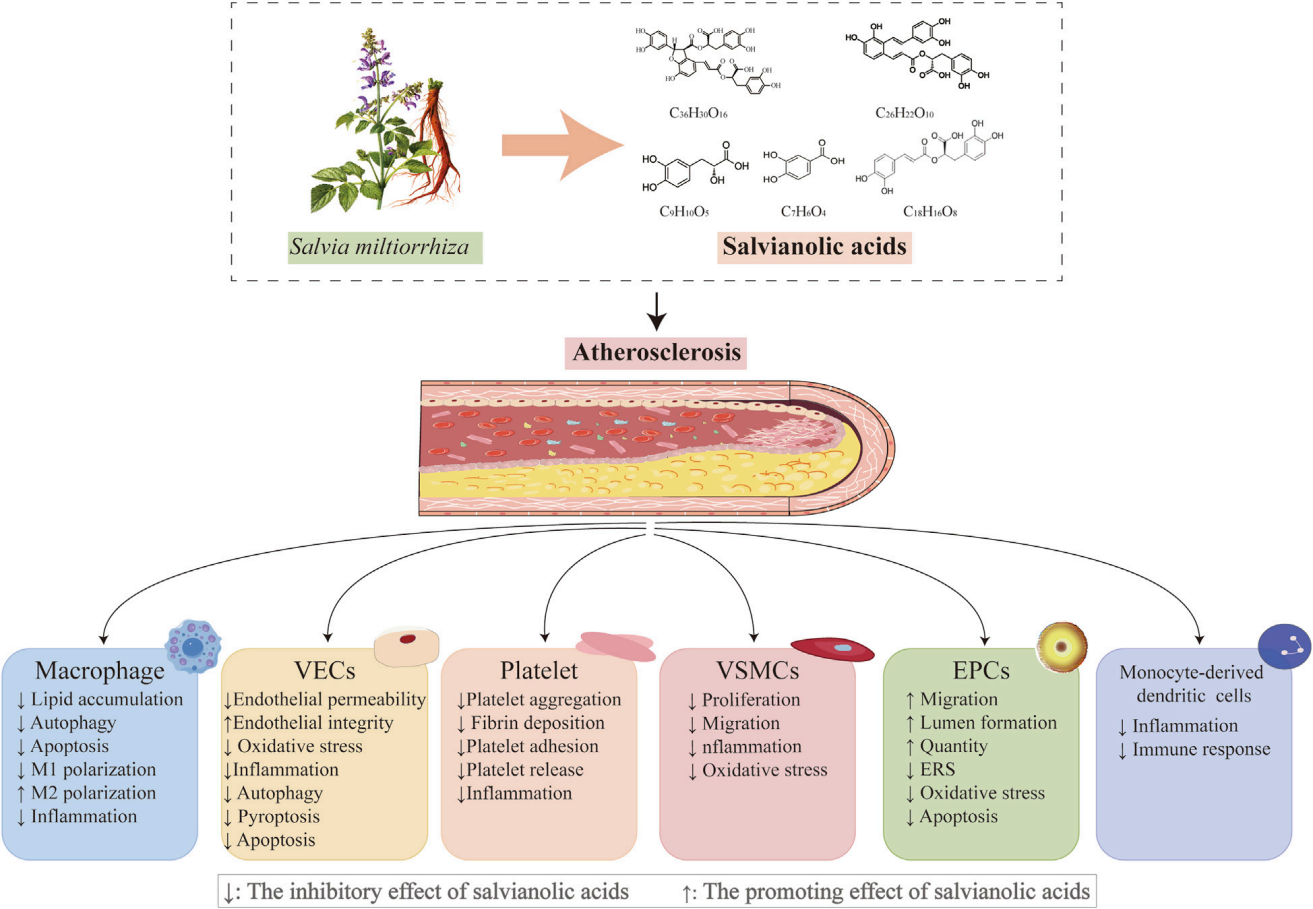
Salvianolic acids regulate important cellular processes in atherosclerosis; plant morphology of *Salvia miltiorrhiza* Bunge, whose major water-soluble active metabolites and their chemical structures mediate vasoprotective actions (chemical structure of danshensu (PubChem CID: 11600642), molecular formula: C_9_H_10_O_5_; chemical structure of salvianolic acid A (PubChem CID: 5281793), molecular formula: C_26_H_22_O_10_; chemical structure of salvianolic acid B (PubChem CID: 6451084), molecular formula: C_36_H_30_O_16_; chemical structure of rosmarinic acid (PubChem CID: 5281792), molecular formula: C_18_H_16_O_8_; chemical structure of protocatechuic acid (PubChem CID: 72), and molecular formula: C_7_H_6_O_4_).

The water-soluble metabolites of *Salvia miltiorrhiza* Bunge [Lamiaceae; Salviae miltiorrhizae radix et rhizoma], particularly the salvianolic acid analogs (including danshensu (DSS), salvianolic acid A (SAA), salvianolic acid B (SAB), rosmarinic acid (RA), and protocatechuic acid (PCA)) (Figure 2), have garnered significant attention in the prevention and treatment of AS owing to their well-defined pharmacophoric properties and comprehensive mechanisms (Duan et al., 2021; Li et al., 2018; Qin et al., 2015; Yang et al., 2019; Zhang et al., 2021a; Zhang et al., 2021b; Zhou et al., 2005a). Research has demonstrated that salvianolic acid exerts multifaceted protective effects on endothelial cells through multiple pathways, including the suppression of yes-associated protein (YAP)/transcriptional coactivator with PDZ-binding motif (TAZ)/c-Jun N-terminal kinase (JNK)-mediated endothelial inflammation, inhibition of NLR family pyrin domain containing 3 (NLRP3)-associated endothelial pyroptosis, modulation of silent mating type information regulation 2 homolog-1 (SIRT1)-mediated oxidative stress and autophagy, and regulation of mitogen-activated protein kinase (MEK)/extracellular signal-regulated kinase (ERK)-associated endothelial apoptosis. Notably, salvianolic acid exhibits targeted therapeutic effects on the key pathological processes of AS, including vascular smooth muscle cell (VSMC) proliferation and migration, macrophage lipid metabolism regulation, endothelial progenitor cell differentiation, and platelet activation inhibition (Jia et al., 2019; Li et al., 2018; Xu et al., 2018b). These multicellular multitarget synergistic mechanisms present distinct advantages over conventional single-target synthetic drugs. Furthermore, salvianolic acid demonstrates significant pharmacokinetic interactions, reducing statin clearance by 43% (to 57% of the original levels) and enhancing irbesartan plasma concentration by 49.9%, thereby highlighting its potential in combination therapy.

Hence, the present work focuses on the multidimensional anti-AS mechanisms of salvianolic-acid-based components, revealing their integrated regulatory properties that distinguish them from existing single-target drugs by systematically resolving their regulatory networks on vascular endothelial cells (VECs), macrophage polarization, and platelet activation. By combining these with clinical evidence, toxicity evaluations, pharmacokinetic data, and drug–drug interactions, we comprehensively evaluate the academic and application prospects of salvianolic acid in the prevention and treatment of AS in the current era of precision medicine. In addition to highlighting the therapeutic potential and safety of salvianolic acid in AS, we address the limitations of the current review and discuss some priorities for future research with the aim of providing a comprehensive perspective for further studies.

## 2 Research methodology

**FIGURE 3:**
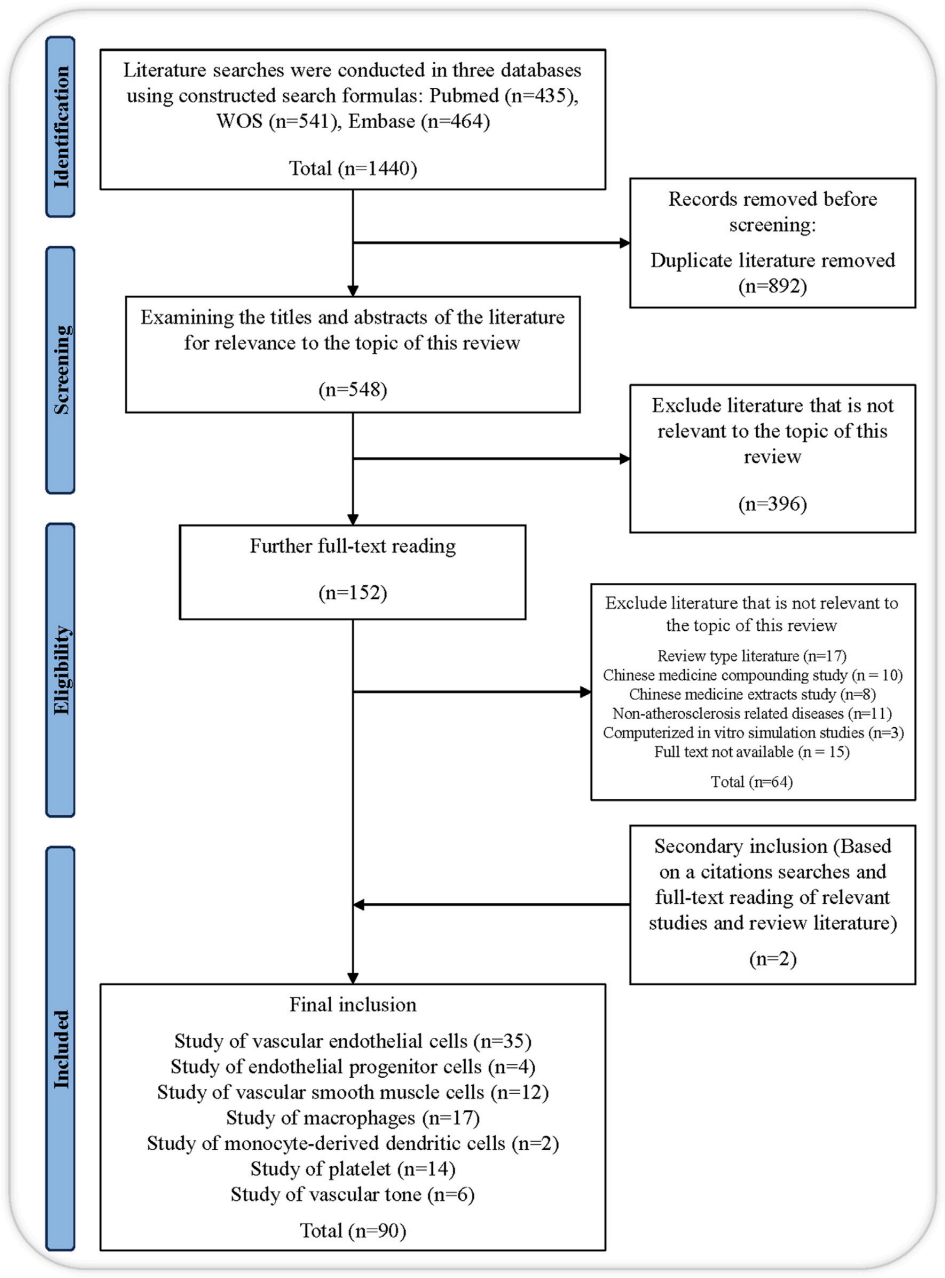
Flow chart of this study.

We conducted a literature search of three scientific databases, namely, Web of Science, PubMed, and Embase, without using filters and applied the following keywords in appropriate combinations to obtain the relevant search formula: “salvianolic acid,” “salvianolic acid A,” “salvianolic acid B,” “danshensu,” “rosmarinic acid,” “lithospermic acid,” “protocatechualdehyde,” “protocatechuic acid,” “salvianolic acid C,” “atherosclerosis,” “anti-atherosclerotic effect,” “vascular endothelial cell,” “vascular smooth muscle cell,” “leukocyte,” “monocytes,” “macrophages,” “endothelial progenitor cell,” “mast cell,” “neutrophils,” “platelet.” The therapeutic roles and mechanism investigations of salvianolic acid in AS are the main emphasis of this review. To eliminate articles unrelated to our study subject, we reviewed the titles, abstracts, and full text of works published over the previous 24 years. Parallelly, we conducted a relevancy search to find references to pertinent research and literature reviews. A total of 90 references pertaining to the review topic were ultimately included after eliminating duplicates using the automated and manual duplicate identification technique of Endnote 20 (Figure 3).

## 3 Salvianolic acids and atherosclerotic vascular disorders: emerging action mechanisms and cellular targets

**FIGURE 4:**
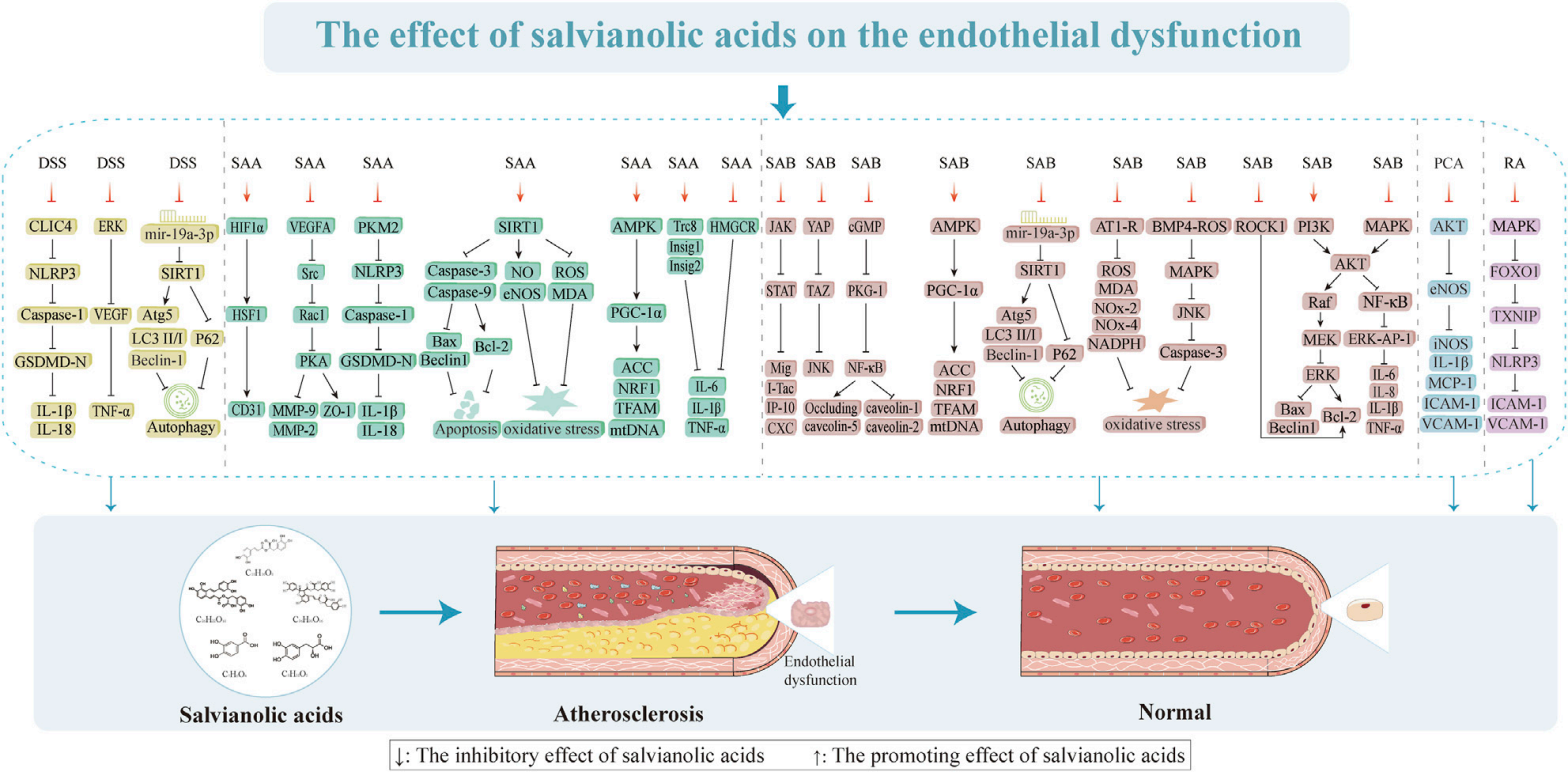
Effects of salvianolic acids on endothelial dysfunction.

Salvianolic acid is the water-soluble active ingredient of *S. miltiorrhiza* Bunge and shows increasing evidence of vasoprotective properties (Ma et al., 2020; Xu et al., 2018a; Zhao et al., 2023). Salvianolic acid is notable for its anti-atherosclerotic actions in a range of animal and cellular models linked with AS, such as VECs, VSMCs, platelets, macrophages, and endothelial progenitor cells (EPCs) (Ma et al., 2022; Tang et al., 2022; Xie et al., 2023; Zeng et al., 2023; Zhang et al., 2024a) (Table 1). By altering several of these biological processes, salvianolic acid slows the progression of AS (Figure 4). Furthermore, salvianolic acid reduces changes in vascular tone caused by mechanical stimulation via altering calcium ion transport and blood flow shear, indicating that it may have additional benefits in treating AS (Pan et al., 2022). Crucially, salvianolic acid protects against AS at various stages of its development. During the initial phases of AS, salvianolic acid protects by reducing endothelial damage; as the disease advances, crucial elements of AS development, including platelet activation, macrophage polarization, and VSMC migration and proliferation, are also inhibited by salvianolic acid (Sun et al., 2012; Zhao et al., 2020; Zhou et al., 2020). To better demonstrate the anti-atherosclerotic properties of salvianolic acid, we explore the cellular pathways linked with AS and the precise action mechanisms of salvianolic acid in these pathways. Understanding these processes will help us better appreciate and utilize the potential of salvianolic acid in treating AS.

**TABLE 1:** Anti-atherosclerotic effects of salvianolic acids.

Cellular targets	Salvianolic acids	Model	Dosage	Duration	Described effects	Mechanisms	Reference
Vascular endothelial cells (VECs)	DSS	HUVECs TNF-α (100 ng/mL)	0, 10, 20, 50 μg/mL	12 h	ROS↓, VEGF↓, ERK↓, VE-cadherin↓	Attenuates increased endothelial permeability	Ding et al. (2005)
		HUVECs TNF-α (100 ng/mL)	10, 20 μg/mL	12 h	VE-cadherin↓, β-catenin↓	Inhibits TNF-α-induced increase in tyrosine phosphorylation	Ding and Yuan (2007)
		SD rats; cerebral artery occlusion/reperfusion	7.5, 30 mg/kg	3 d	NLRP3↓, ASC↓, caspase-1↓, IL-1 β↓, IL-18↓, GSDMD-N↓, CLIC4↓	Inhibits CLIC4/NLRP3 inflammasome-mediated endothelial cell pyroptosis	Zhang et al. (2024b)
		bEnd.3; oxygen-glucose deprivation model	12.5, 25, 50 μM	—			
		HUVECs H_2_O_2_ (200 μM)	0.1, 0.25 μg/mL	24 h	ROS↓, GSH/GSSG↑, SOD↑, LC3 I/II↓, Beclin-1↓, P62↑, SIRT1↓, miR-19a-3p↓	Inhibits autophagy via mir-19a/SIRT1 pathway	Guo et al. (2021)
	SAA	Spontaneously hypertensive rats	2.5, 5, 10 mg/kg/d	4 weeks	MMP-9↓	Inhibits the remodeling of vessels and normalized endothelial dysfunction	Teng et al. (2016)
		HUVECs LPS (10 μg/mL)	0.01, 0.1, 1.10 μmol/L	24 h			
		SD rats; autologous thrombus stroke model	10 mg/kg	5 d	MMP-9↓, MMP-2↓, ZO-1↑, claudin-5↑, occluding↑, VE-cadherin↑, HIF-1α↓, VEGFA↓, Src↓, Rac1↓, PAK↓	Prevents endothelial injury by inhibiting the Src signaling pathway	Liu et al. (2021a)
		HBMECs; oxygen-glucose deprivation model	10 μM	—			
		ApoE^−/−^ mice; high-fat diet (HFD)	20, 40 mg/kg	12 weeks	HMGCR↓, Trc8↑, Insig1↑, Insig2↑, ubiquitinated HMGCR↑, IL-1β↓, IL-6↓, TNF-α↓	Inhibits inflammation through Trc8-regulated degradation	Xie et al. (2023)
		HUVECs; ox-LDL (100 μg/mL)	50, 100, 200 μM	24 h			
		ApoE^−/−^ mice; STZ (50 mg/kg), western diet with 0.5% cholesterol	10, 20 mg/kg	4 weeks	Caspase-1↓, GSDMD - N↓, NLRP3↓, ASC↓, PKM2↓, PKR↓	Regulates pyroptosis of endothelial cells by directly targeting PKM2	Zhu et al. (2022)
		HUVECs HG (33 mM)	5, 10, 25 μM	0.5 h			
		HUVECs HG (30 mM)	100, 200, 400 mg/L	4, 12, 24 h	ROS↓, Sirt1↑, p-eNOS↓	Inhibits vascular endothelial dysfunction by modulating the Sirt1-eNOS pathway	Zhai et al. (2019)
		Wistar rats HFD, STZ (30 mg/kg)	1 mL/kg	10 weeks	von Willebrand factor↓, MDA↓, eNOS↑, AGEs↓	Suppresses oxidative stress and age-induced endothelial dysfunction	Yang et al. (2011)
		EAhy926 AGEs (10%)	10^–8^,10^–7^,10^–6^, 10^–5^ mol/L	2 h			
		HUVECs HG (30 mM)	100, 200, 400 mg/L	4, 12, 24 h	Bcl-2↑, Bax↓, Caspase-3↓, Caspase-9↓, Sirt1↑, p-eNOS↓	Inhibits vascular endothelial dysfunction by modulating the Sirt1-enos pathway	Zhai et al. (2019)
		HUVECs; AMPK inhibitor compound C (2.5 μM), AICAR (0.5 mM)	0.001, 0.01, 0.1 μM	24 h	mtDNA↑, PGC-1α↑, NRF1↑, TFAM↑, AMPK↑, ACC↑	Promotes mitochondrial biosynthesis and improves mitochondrial function via activation of the AMPK/PGC-1α signaling pathway	Wang et al. (2022)
	SAB	HUVECs TNF-α (100 ng/mL)	0, 10, 20, 50 μg/mL	12 h	ROS↓, VEGF↓, ERK↓, VE-cadherin↓	Attenuates increased endothelial permeability	Ding et al. (2005)
		HUVECs TNF-α (100 ng/mL)	10, 20 μg/mL	12 h	VE-cadherin↓, β-catenin↓	Inhibits TNF-α-induced increase in tyrosine phosphorylation	Ding and Yuan (2007)
		C57BL/6J mice; transverse aortic constriction	200 mg/kg	6 weeks	CD31↑, HSF1↑, HIF1α↑	Protects endothelial cells against pressure overload via the HIF1α/HSF1/CD31 pathway	Li et al. (2022)
		ApoE^−/−^ mice; HFD	30 mg/kg	4 weeks	YAP↓, TAZ↓, JNK↓, NF-κB P65↓, Total P65↓, TNF-α↓, p-YAP↑, p-TAZ↑, ROS↓, MDA↓, SOD↑, GPX↑, IL-6↓, IL-1β↓	Ameliorates atherosclerosis by inhibiting the YAP/TAZ/JNK signaling pathway	Yang et al. (2020b)
		ECs; ox-LDL (30 μg/mL)	30 μg/mL	12 h			
		EAhy926; ADP	1, 5, 10 μg/mL	24 h	NF-κBp65↓, ICAM-1↓, IL-1 β↓, IL-6↓, IL-8↓, MCP-1↓	Attenuates platelet-mediated inflammatory responses by inhibiting NF-κB	Xu et al. (2015)
		HAECs; TNF-α (1, 2, 4 ng/mL)	1, 2.5, 5, 10, 20 μg/mL	18 h	VCAM-1↓, ICAM-1↓, NF-κB↓	Attenuates VCAM-1 and ICAM-1 expressions	Chen et al. (2001)
		BAECs INF-γ (10 ng/mL)	1, 10, 25, 50 μM	6 h	JAK2↓, STAT1↓, IP-10↓, CXC↓, Mig↓, I-Tac↓	Suppresses JAK-STAT signaling pathways and reduces CXC chemokine secretion	Chen et al. (2011)
		HUVECs TNF-α (10 ng/mL)	0.017, 0.05, 0.15 μM	12, 18 h	PAI-1 mRNA↓, ERK-AP-1↓, NF-κB↓	Inhibits PAI-1 mRNA expression through inhibition of the ERK-AP-1- and NF-κB-dependent pathways	Zhou et al. (2005b)
		C57BL/6J mice; Ang II (1 μM)	25 mg/kg/d	11 d	ROS↓, NOx-2↓, NOx-4↓, AT1 receptor↓, nitrotyrosine↓, NADPH subunits↓	Inhibits AT1 receptor stimulated NADPH oxidase and associated ROS	Ling et al. (2017)
		SD rats; HFD, STZ (40 mg/kg)	80, 160 mg/kg	6 weeks	NO↑, eNOS↑, NOX2↓, MDA↓	Suppresses endothelial cell apoptosis and enhances endothelium-derived NO production	Ren et al. (2016)
		db/db mice	10 mg/kg	1 week	BMP4↓, ROS↓, p38 MAPK↓, JNK↓, caspase-3↓	Ameliorates endothelial dysfunction by breaking the BMP4-ROS cycle and inhibiting p38 MAPK/JNK/caspase-3	Liu et al. (2021b)
		rCMECs H_2_O_2_ (200 μM)	10, 20, and 100 μM	—	cleaved caspase-9↓, ERK↓, AKT↑, PI3K↑, Raf↑, MEK↑	Protects rCMECs against apoptosis through the PI3K/AKT/Raf/MEK/ERK pathway	Liu et al. (2007)
		HUVECs; ox-LDL (100 μg/mL)	3.125, 6.25, 12.5, 25, 50, and 100 μg/mL	12 h	IL-6↓, TNF-α↓, MCP-1↓, p38MAPK↓, AKT↑, NF-κB↓, Bax↓, Smac↓, Bcl-2↑, cIAP-2↑, SOD↑, GSH-Px↑, CAT↑, LOX-1↓, NOX4↓	Protects against endothelial apoptosis through antioxidative and anti-inflammatory mechanisms	Yang et al. (2018)
		HUVECs; ox-LDL	5, 10, and 20 μg/mL	24 h	SOD↑, MDA↓, p53↓, Bcl-2/Bax↑, caspase-3↓	Exhibits antiapoptotic effects by suppressing oxidative stress, p53, and caspase-3	Chen et al. (2017)
		EAhy926; HG (25 mM), ox-LDL (100 μg/mL)	1, 10, 50, and 100 μM	24 h	LOX-1↓, mtROS↓, cleaved caspase-8↓, p-DRP1↓, FIS1↓, ROCK1↓	Reduces apoptosis-related proteins and fission proteins through suppression of the ROCK1-mediated pathway	Ko et al. (2020)
		SD rats; HFD, STZ (40 mg/kg)	80 and 160 mg/kg	6 weeks	Bcl-2↑, Bax↓	Suppresses endothelial cell apoptosis and enhances endothelium-derived NO production	Ren et al. (2016)
		db/db mice	50 mg/kg	6 weeks	Bcl-2↑, Bax↓, Beclin1↓, Parkin↓, Pink1↓	Improves endothelial and mitochondrial dysfunction by downregulating apoptosis and mitophagy of endothelial cells	Xiang et al. (2022)
		HUVECs HG (30 mM)	0, 5, 10, 30, 50, 80, and 100 μM	48 h			
		ICR mice; isoproterenol (3 mg/kg)	10 mg/kg	1 week	VEGF↑, PDGF↑, Bcl-2↑, Bax↓, LDH↓, MDA↓, SOD↓, Beclin1↑, Atg5↑, P62↓, LC3 II/LC3 I↑	Inhibits apoptosis and promotes angiogenesis by regulating autophagy	Chen et al. (2023)
		HUVECs VEGF (40 ng/mL)	20, 40, and 80 μM	24 h			
		HUVECs H_2_O_2_ (200 μM)	0.25 and 0.5 μg/mL	24 h	ROS↓, GSH/GSSG↑, SOD↑, LC3 I/II↓, Beclin-1↓, P62↑, SIRT1↓, miR-19a-3p↓	Inhibits autophagy via the mir-19a/SIRT1 pathway	Guo et al. (2021)
		db/db mice	50 mg/kg	6 weeks	Beclin1↓, Parkin↓, Pink1↓	Improves endothelial and mitochondrial dysfunctions by downregulating apoptosis and mitophagy of endothelial cells	Xiang et al. (2022)
		HUVECs HG (30 mM)	0, 5, 10, 30, 50, 80, and 100 μM	48 h			
		HUVECs LPS (10 μg/mL)	100 μM	16 h	eNOS↓, NO↓	Promotes endothelium-dependent vasodilation	Joe et al. (2012)
	RA	EA.hy926; HG, ox-LDL	1, 10, 50, and 100 μM	24 h	ICAM-1↓, VCAM-1↓, ROS↓, p38 MAPK↓, FOXO1↓, TXNIP↓	Modulates ROS/p38 MAPK/FOXO1/TXNIP/NLRP3 activation to inhibit monolayer leakage of VECs	Nyandwi et al. (2022)
	PCA	Microvascular endothelial cells; IL-1β	1, 10, and 100	—	ICAM-1↓, VCAM-1↓, p-AKT/AKT, p-eNOS/NOS↓, iNOS↓, MCP-1↓, IL-1β↓	Ameliorates endothelial dysfunction by enhancing the AKT/eNOS pathway	Chook et al. (2023)
Vascular smooth muscle cells (VSMCs)	SAA	SD rat VSMCs PDGF-BB (40 ng/mL)	0.003, 0.01, 0.03 and 0.1 μM	2 h	OPN↓, VCAM-1↓, ICAM-1↓, CDK2↓, cyclin E↓, p27↑, PDGFRβ↓, ERK1/2↓	Inhibits migration and proliferation of VSMCs via the PDGFRβ-ERK1/2 cascade	Sun et al. (2012)
		HUASMCs, HASMCs; PDGF-BB (20 ng/mL)	0.1, 0.3, and 1.0 μM	2 h	p21↓, cAMP↑, PKA↑, CREB↑	Prevents neointimal hyperplasia via the camp/PKA/CREB cascade	Sun et al. (2016b)
		Mouse aortic VSMCs; Ang II (10^–7^ mol/L)	2.5, 5, 10 μM	3, 6.12 h	KLF5↓, cyclin D1↓, PCNA↓, miR-146a↓	Inhibits VSMC proliferation and intimal hyperplasia by downregulating mir-146a expression	Zhao et al. (2019)
		C57BL/6 mice; carotid ligation	7 mg/kg/d	3 d			
	SAB	HASMCs LPS (100 ng/mL)	2.5, 5, and 10 μM	24 h	COX-2↓, PGE_2_↓, ICAM-1↓, ERK↓, JNK↓, NADPH oxidase↓, p47^phox^↓	Inhibits the expression COX-2 to decrease intimal hyperplasia	Chen et al. (2006)
		ApoE^−/−^ mice	0.3%	12 weeks			
		Thoracic aortic VSMCs; SDF-1α (10 ng/mL)	0.075 mg/mL	—	CXCR4↓, ERK1/2↓, MAPK↓, Raf-1↓, MEK↓, FAK↓, NF-κB↓	Blocks SDF-1α/CXCR4-induced cell proliferation and migration	Pan et al. (2012)
		HASMCs; LPS (10–100 ng/mL)	5, 10, and 20 μM	12, 24, 48 h	MMP-2↓, MMP-9↓, ERK↓, JNK↓	Suppresses migration of VSMCs	Lin et al. (2007)
		Mouse aortic VSMCs; HG (25 mM)	2.5, 5.10 μM	6, 12, 24 h	IL-1β↓, TNF-α↓, FOXO1↓, miR-486a-5p↑	Modulates the mir-486a-5p/FOXO1 axis to alleviate inflammation of VSMCs	Zhang et al. (2024a)
		C57BL/6mice; high-calorie diet, STZ	7 mg/kg	16 weeks			
		VSMCs PDGF (20 ng/mL)	1, 5, 10, 50, and 100 μM	24 h	ROS↓, NADP/NADPH↓, HO-1↑, AKT↓, Nrf2↑	Activates the Nrf2-HO-1 antioxidant signaling pathway	Lee et al. (2014)
	RA	Human aortic VSMCs	25, 50, and 100 μM	24 h	HO-1↓, NQO1↓, GCLM↓, GST↓, Nrf2↑, NF-κB↓	Inhibits proliferation, migration, and phenotypic transformation of VSMCs	Chen et al. (2022a)
		Arterial smooth muscle cells	5, 50, and 100 μM	24 h	CRP↓, ROS↓, NLRP3↓, ASC↓, Caspase-1↓, IL-18, IL-1β	Inhibits the ROSNLRP3-CRP axis	Yao et al. (2019)
		Murine aortic SMC	50, 100, 200, and 400 μM	24 h	TNF-α↓, IL-8↓, iNOS↓, NO↓, Erk↓, JNK↓, p38 MAPK↓, NF-κB↓	Ameliorates the inflammatory responses of VSMCs by inhibiting MAPK/NF-κB signaling	Chen et al. (2022b)
	PCA	Cell line A7r5	50–150 μg/mL	48 h	AKT↓, p-AMPK↑, p53↑, p21Cip1↑, cyc↓	Suppresses cell proliferation and cell cycle progression of VSMCs by activating AMPK	Lin et al. (2015)
Endothelial progenitor cells (EPCs)	DSS	SD rats; left coronary artery ligation	60 mg/kg	2 weeks	VEGF↑, bFGF↑, SDF-1α↑, CXCR4↑	Promotes the proangiogenesis functions of EPCs for migration and tube formation through the SDF-1α/CXCR4 axis	Yin et al. (2017)
		Bone-marrow-derived EPCs; hypoxia and serum starvation	10 μM	24 h			
	SAA	SD rats; left coronary artery ligation	2.5, 5.0, and 10 mg/kg	1 week	VEGF↑, VEGFR-2↑, MMP-9↓	Promotes the numbers and functions of EPCs	Li et al. (2014)
		EPCs from spleen tissue	—	—			
		Bone-marrow-derived EPCs; H_2_O_2_ (1 mM)	5, 10, 20, and 40 μM	24, 48, 96 h	p-AKT↑, p-mTOR↑, p-p70S6K↑, p-4EBP1↑, ROS↓, Bax/Bcl-xL↓, Cyt C↓, Caspase-3↓, MKK3/6↓, ATF2↓, Nox4↓, eNOS↓, NADPH oxidase↓, p38 MAPK↓, ERK1/2↓	Promotes the migratory and tube formation abilities of EPCs via modulation of mTOR-4EBP1 and MKK3/6-p38 MAPK-ATF2	Tang et al. (2014)
	SAB	Human BM-EPCs; tunicamycin (5 μg/mL)	10, 20 μM	48 h	ROS↓, SOD2↑, GRP78↓, ATF4↓, eIF-2α↓, Rac1↓, ATF2↓, IL-1β↓, IL-18↓, NLRP3↓, TXNIP↓, AMPK↓, Foxo4↓, KLF2↓	Suppresses pyroptosis and endoplasmic reticulum stress via AMPK/foxo4/KLF2 and Syndecan-4/Rac1/ATF2 signaling in EPCs	Tang et al. (2022)
Platelets	DSS	SD rats; FeCl_3_	30 mg/kg/d	1 week	CD62p↓, SIRT1↑, ROS↓, mtDNA↓, DC-SIGN↓	Obstructs platelet activation via the SIRT1/ROS/mtDNA pathway	Xue et al. (2022)
		Platelets from SD rats	1, 10, 50, 100, 500 μM	—			
		SD rats; arteriovenous shunt, venous thrombosis model	15, 30, 60 mg/kg/d	1 week	COX-2↓, TXB2↓, 6-keto-PGF_1α_↑	Normalizes TXA2/PGI2 balance to show antithrombotic effects and antiplatelet aggregation	Yu et al. (2014)
	SAA	SD rats; arteriovenous shunt model	2.5, 5, 10, and 20 mg/kg/d	5 d	cAMP↑, blood viscosity↓, plasma viscosity↓, hematocrit↓	Possesses antithrombotic activities and may involve cAMP	Fan et al. (2010)
		Washed platelets from rats and humans	5, 200, 400, 600, 800, and 1,000 μg/mL	—			
		LDLr^−/−^ mice; intravenous administration	10 mg/kg	—	p-AKT^Ser473/474^↓, Rap 1↑, platelet fibrinogen↓, P-selectin↓	Inhibits platelet activation by inhibiting PI3K	Huang et al. (2010)
		Washed platelets from humans	0.02, 0.05, and 0.1 mg/mL	10 min			
		SD rats; ischemia-reperfusion injury	10 mg/kg	—	p-selectin↓, TNF-α↓, IL-1β↓, NO↑, platelet aggregation rate↓	Reduces platelet activation and inflammation	Yuan et al. (2017)
	SAB	Platelets and immobilized collagen	250, 500, 750, and 1,000 μg/mL	—	α2β1↓	Inhibits platelet adhesion to collagen by interfering with the collagen receptor α2β1	Wu et al. (2008)
		C57BL/6J mice; ferric chloride	15 mg/kg	—	platelet aggregation↓, P-selectin↓, RCA-1↓	Suppresses thrombosis through both thrombin-dependent and thrombin-independent pathways	Neves et al. (2024)
		Human platelets	0.01, 0.02, 0.04, 0.08, and 0.16 mM	—			
	PCA	Human gel-filtered platelets; H_2_0_2_	0, 0.25, 0.5, and 1.0 μM	50 min	Bax↓, Bcl-2↓, P13K↓, AKT↓, GSK3β↓	Protects platelets from oxidative-stress-induced apoptosis by downregulating ROS-mediated PI3K/AKT/GSK3β signaling	Ya et al. (2021)
		Human platelets	1, 5, 10, 25 μM	—	SIPA↓, platelet activation↓, vWF-GP Ib interaction↓	Protects against high-shear-induced platelet aggregation by blocking vWF-GP Ib interaction	Kim et al. (2012)
	RA	New Zealand white rabbits	1, 3, 10, 30, 100 μM	5 min	Platelet aggregation↓, Erp57↓	Inhibits platelet aggregation possibly by inhibiting ERp57	Zou et al. (2018)
Macrophages	DSS	Bone-marrow-derived macrophages; Pam3CSK4	2.5, 5, 10 μM	—	IL-6↓, IL-12↓, p65↓	TLR2 and macrophages are affected through the NF-κB signaling pathway	Ye et al. (2020)
		ApoE^−/−^ mice; HFD	40, 80 mg/kg	—	IL-6↓, TNF-α↓, IL-1β↓, IKKβ↓, IKKγ↓, p65↓, p-IKKβ↓, p-IKKβ/IKKβ↓, p-IκBα↓, p-IκBα/IκBα↓, p-p65↓, p-p65/p65↓, TLR4↓	Stabilizes vulnerable plaques and suppresses inflammatory responses by inhibiting the NF-κB pathway by targeting IKKβ	Zeng et al. (2023)
		Macrophage J774A.1; ox-LDL	12.5, 25, 50, 100 μM	24 h			
	SAA	Peritoneal macrophages Ang II (1 μM)	12.5, 25, 50 μM	12 h	Bax↓, Bcl-2↑, p-NF-κBp65↓, p-AKT473↓, p-AKT308↓, ROS↓, MDA↓, SOD↑	Exhibits antioxidant and antiapoptotic effects via the NF-κB and AKT pathways	Li et al. (2016)
		ApoE^−/−^ mice; HFD	5 mg/kg	12 weeks	p38↓, CCL-20↓, CCR6↓	Inhibits progression of atherosclerotic plaques and lipid accumulation	Zhang et al. (2014)
		RAW264.7 cells TNF-α (100 ng/mL)	1, 10, 100 mg/mL	2 h			
	SAB	THP-1 macrophages; LPS (1 μg/mL)	1, 5, 25 μM	1 h	IL-1β↓, IL-6↓, TNF-α↓, p-p65↓, p-IκBα↓, NF-κB↓, TLR4↓	Displays anti-inflammatory properties via the TLR4/NF-κB pathway	Liu et al. (2018a)
		RAW264.7 cells; LPS (1 μg/mL)	0.1, 1, 10, 50, 100, 200 μM	24 h	NO↓, IL-1β↓, IL-6↓, IL-12↓, iNOS↓, IL-10↑, Arg1↑, Mincle↓, Sec62↓, Syk↓, PKCδ↓	Inhibits inflammation through the Mincle-Syk-PKCδ signaling pathway	Li et al. (2021d)
		THP-1 macrophages; phorbol 12-myristate 13-acetate (160 nmol/mL)	0, 0.1, 1, 10 mM	24 h	ABCA1↑, PPAR-g↑, LXRα↑	Promotes expression of ABCA1 and cholesterol efflux through PPAR-g/LXRα	Yue et al. (2015)
		RAW264.7 cells; LPS (1 μg/mL)	100, 200, 400 μM	12 h	HO-1↑, NO↓, iNOS↓, TNF-α↓	Inhibits inflammatory responses in macrophages	Joe et al. (2012)
		RAW264.7 cells; cholesterol crystals (0, 400, 800, 1,200 μg/mL)	0, 40, 80, 120, 160, 200, 240, 280, 320, 360 μM	—	Caspase-3/pro-Caspase-3↓, Bax/Bcl-2↓, TNF-α↓, IL-6↓, p62↓, LC3-II↑, beclin-1↑, p-mTOR/mTOR↓, p-AKT/AKT↓	Improves macrophage autophagy dysfunction by inhibiting the AKT/mTOR signaling pathway to attenuate macrophage apoptosis	Sun et al. (2021)
		THP-1 cells from human, RAW264.7 cells, primary macrophages; DiI-acLDL (2 μg/mL)	1, 10,100 μM	4 h	CD36↓	Reduces oxidized-LDL-induced CD36 gene expression	Bao et al. (2012)
		RAW264.7 cells; LPS (100 ng/mL), IFN-γ (2.5 ng/mL)	0, 50, 100, 150, 200, 250, 300, 350 μM	24 h	iNOS↓, TNF-α↓, IL-6↓, Arg-1↑, IL-10↑, beclin-1↑, p62↓, NF-κB p65↓, LC3-II↑, p-AKT↓, p-mTOR↓	Inhibits NF-κB signaling pathway activation and downregulates AKT/mTOR activation to promote autophagy	Zou et al. (2022)
		Bone-marrow-derived macrophages; LPS (100 ng/mL)	5, 10, 20 μM	24 h	IL-6↓, IL-1β↓, iNOS↓, CCL2↓, CCL5↓, CCR2↓, TNF-α↓, Arg1↑, Clec10a↑, Mrc↑, IL-10↑, mTORC1↓, p-S6↓, p-p70s6k↓	Shifts macrophage polarization from M1 to M2 in MI/R hearts by inhibiting mTORC1-dependent glycolysis	Zhao et al. (2020)
		Mice; ligation of the left anterior descending artery	80 mg/kg	3 d 7 d			
		C57BL/6J; hind limb ischemia model	15 mg/kg/d	12 d	IL-6↓, SIRT1↑, PI3K↑, AKT↑	Improves limb ischemia via M2 macrophage polarization by the SIRT1/PI3K/AKT pathway	Niu et al. (2022)
		Bone-marrow-derived macrophages; IL-4 (10 ng/mL)	25 μM	24 h			
		LDLR^−/−^ mice; HFD	25 mg/kg	12 weeks	TC↓, TG↓, LDL-C↓, IL-1β↓, IL-6↓, TNF-α↓, JNK↓, ERK1/2↓, p38↓, IκB↓, NF-κB↓, iNOS↓, VCAM↓, p-JNK↓, p-ERK1/2↓, p-P38↓, p-IκB↓, p-NF-κB↓	Exerts anti-inflammatory effects on atherosclerosis via the MAPKS/NF-κB signaling pathway	Zhang et al. (2022)
		RAW264.7 cells; ox-LDL, LPS (100 μg/mL)	1.25, 2.5 5 μg/mL	—			
	RA	THP-1 monocytes; HG, ox-LDL	100 μM	24 h	LOX-1↓, CD36↓, ABCA1↑, ABCG1↑, JAK2↑, STAT3↑	Increases macrophage cholesterol efflux by regulating ABCA1 and ABCG1	Nyandwi et al. (2021)
	PCA	J774A.1 cells; ox-LDL	25 μM	24 h	ROS↓, Caspase-3↓, Bax↓, Nrf2↑, JNK↑, p38↓, p53↑, p38 MAPK↓, Pkc δ↓	Prevents oxidized-LDL-induced apoptosis by activating JNK/Nrf2 survival signals	Vari et al. (2015)
		ApoE^−/−^ mice	15 mg/kg	14 weeks	iNOS↓, IL-6↓, TNF-α↓, CD206↑, Arg-1↑, IL-10↑, MHC II↓, CD11c↓, NF-κB↓, PI3K↓, AKT↓, SOCS1↓, CD163↑, KLF4↑	Alleviates atherosclerosis by inhibiting M1 polarization and promoting M2 activation	Liu et al. (2019)
		Mouse macrophage cell line	10, 20, 50, 100 μM	—			
		THP-1 cells from humans	0.25, 0.5, 1 μM	—	MerTK↑, KLF4↑, miR-10b↑	Promotes continual efferocytosis of macrophages by activating KLF4-MerTK	Li et al. (2023a)
		C57BL/6J	5 mg/kg	1 week			

### 3.1 Restoration of endothelial function

VECs are essential for preserving both the vascular function and integrity of the internal environment (Xu et al., 2021). Several studies have demonstrated that endothelial dysfunction and injury occur frequently before vascular wall structural lesions and are the causes of AS with strong links to its pathological development (Libby et al., 2011). It has been proposed that endothelial dysfunction typified by hyperpermeability, increased oxidative stress, and chronic inflammation is a hallmark of several human vascular illnesses, including diabetes mellitus, hypertension, and AS, that affect the development and progression of AS (Davignon and Ganz, 2004; Tamargo et al., 2023; Xu et al., 2021). The atherosclerotic process is initiated by endothelial barrier dysfunction, which then facilitates monocyte infiltration and triggers localized inflammation that are subsequently amplified through nuclear factor kappa-B (NF-κB) pathway activation to exacerbate oxidative stress. This pathological cascade not only induces direct mitochondrial structural damage but also establishes a self-perpetuating “inflammation and oxidative stress” cycle mediated by NLRP3 inflammasome activation (Tanase et al., 2023). Progressive mitochondrial dysfunction disrupts cellular metabolism and energy homeostasis, initially stimulating protective autophagy as a compensatory mechanism (Li et al., 2021a). However, persistent damage ultimately results in autophagic flux impairment and subsequent accumulation of dysfunctional mitochondria, which concurrently activate both the apoptotic and pyroptotic cell death pathways. Consequently, AS progresses from the initial lipid-driven phase (characterized by autophagy-mediated protection) to the advanced vulnerable plaque stage (marked by pyroptosis-dominated inflammatory destruction), establishing a pathogenic cascade of endothelial dysfunction, metabolic dysregulation, and cell death activation. Thus, we explain the various aspects of endothelial dysfunction as well as the action mechanisms and intervention targets of salvianolic acids to improve endothelial dysfunction.

#### 3.1.1 Improvement of endothelial permeability and integrity

The structural integrity of the vascular endothelium is vital for sustaining vascular function and providing a non-thrombogenic lining for the cardiovascular system (Abdelsalam et al., 2019). When the cellular connection is compromised, cell permeability increases and numerous substances like low-density lipoproteins (LDLs) can flow through the endothelial barrier. Cytoskeletal changes result in cell shrinkage, which increases the cell gap. Cellular tight junction composition, cytoskeleton development, and epithelial cell polarity are all significantly influenced by zonula occludens-1 (ZO-1) (Beutel et al., 2019). Liu et al. (2021a) investigated the effects of various concentrations of SAA (1, 3, and 10 μmol/L) on oxygen-glucose deprivation (OGD)-induced human brain microvascular endothelial cells (HBMECs; their findings demonstrated that SAA at a concentration of 10 μmol/L exerted protective effects on VECs by modulating the vascular endothelial growth factor A (VEGFA)-proto-oncogene tyrosine-protein kinase Src-guanine nucleotide exchange factor VAV2-ras related C3 botulinum toxin substrate 1 (Rac1)-p21 activated kinase (PAK) signaling pathway. Specifically, this protective mechanism involved the reversal of matrix metalloprotein (MMP) production elevation, suppression of ZO-1 protein degradation, and restoration of cell viability diminished by OGD induction (Liu et al., 2021a). MMP-9 is known to promote VEC permeability by dissolving the extracellular matrix and disrupting the cellular junctions to enhance cell movement and the intercellular gap (Li et al., 2021b). SAA can prevent vascular remodeling, which not only preserves the integrity of the endothelial monolayer but also reduces endothelial activity in rats with endothelial dysfunction. This is directly linked to the suppression of MMP-9 production (Teng et al., 2016). The permeability barrier of endothelial cells is maintained in part by CD31 (Lertkiatmongkol et al., 2016); here, SAA retains the intensity of CD31 by mediating the hypoxia-inducible factor 1 alpha (HIF1α)/heat shock factor 1 (HSF1)/CD31 signaling pathway, which has a protective effect on the endothelial permeability barrier (Li et al., 2022). Tyrosine phosphorylation of connexins, which directly affects the structure of VECs, is another method by which tumor necrosis factor-α (TNF-α) promotes endothelial gap junction development and connexin redistribution that increase endothelial permeability (He et al., 2024; Takei et al., 2024). In contrast, DSS and SAB attenuate VE-cadherin phosphorylation and signaling chaperone β-catenin through the tyrosine phosphorylation signaling pathway (Ding and Yuan, 2007). Research has shown that DSS and SAB prevent TNF-α damage to the VEC barrier by suppressing the activation of extracellular regulated protein kinases (ERKs) and lowering the production of vascular endothelial growth factor (VEGF) (Ding et al., 2005). Additionally, SAB (20 µM) was found to change the endothelial permeability via the cyclic guanosine monophosphate (cGMP)/protein kinase G (PKG)/NF-κB signaling cascade, which involves the paracellular pathway (downregulation of occludin and claudin-5) and transcellular pathway (upregulation of caveolin-1 and caveolin-2). Furthermore, SAB mediates the reactive oxygen species (ROS)/HIF-1α/VEGF and miR-200b/VEGF signaling pathways to ameliorate high glucose (HG)-induced endothelial dysfunction by inducing miR-200 (Ding et al., 2005). SAB also directly inhibits VEGF-induced elevation of vascular endothelial permeability (Qui et al., 2001). RA demonstrates dose-dependent inhibitory effects (1, 10, 50, and 100 µM) on both the expression of adhesion molecules and THP-1 monocyte adhesion to endothelial cells (ECs). These protective mechanisms were achieved through effective modulation of the ox-LDL-mediated ROS/p38 mitogen-activated protein kinase (p38 MAPK)/forkhead box protein O (FOXO)1/thioredoxin-interacting protein (TXNIP)/NLRP3 signaling pathway while simultaneously suppressing endothelial monolayer permeability (Nyandwi et al., 2022). Thus, salvianolic acids may help treat endothelial dysfunction by preserving the integrity of the cytoskeleton and endothelium while enhancing the cell junctions and permeability.

#### 3.1.2 Inhibition of endothelial inflammation

Inflammation is one of the leading causes of lipid deposition and plaque development in AS (Weber et al., 2023). Several investigations have revealed that patients with AS have proinflammatory markers like C-reactive protein (CRP) and interleukin (IL)-6 and that high levels of these mediators may cause damage and dysfunction of the VECs (He et al., 2019). SAA (20 and 40 µM) reduces AS by preventing inflammation via insulin-inducible gene (Insig)1, renal cancer chromosome 8 (Trc8), and Insig2-mediated ubiquitylation and recombinant 3-hydroxy-3-methylglutaryl coenzyme A reductase (HMGCR) degradation in a dose-dependent manner (Xie et al., 2023). This was primarily shown through the stimulation of anti-inflammatory IL-10 expression and suppression of the synthesis of proinflammatory proteins TNF-α, IL-1β, and IL-6. These findings imply that SAA may have anti-inflammatory effects and slow the progression of AS, highlighting the significance of Trc8-mediated HMGCR degradation in anti-atherosclerotic cases. After being stimulated by several inflammatory cytokines, the VECs create plasminogen activator inhibitor type 1 (PAI-1), which is crucial for advancing AS (Gimenez-Bastida et al., 2016; Khoukaz et al., 2020). SAB (0.05 and 0.15 µM) decreases endothelial damage and dysfunction caused by endothelial inflammation via controlling the NF-κB and ERK-activator protein 1 (AP-1) pathways, which in turn suppress TNF-α-induced plasminogen activator inhibitor-1 (PAI-1) mRNA synthesis and protein release in HUVECs after 12 h and 18 h of intervention (90.5% and 74.6% as well as 75.1% and 64.2%), respectively (Zhou et al., 2005b). SAB also increases the endothelial fibrinolytic activity, decreases PAI-1 expression, and upregulates t-PA and TM expressions while reducing leukocyte adherence to blood arteries as well as reducing vascular inflammation by blocking the TNF-α-induced upregulation of vascular cell adhesion molecule 1 (VCAM-1) and intercellular cell adhesion molecule-1 (ICAM-1) expressions through NF-κB (1, 2.5, 5, 10, and 20 μg/mL of SAB); VCAM-1 notably attenuates the dose-dependent lowering of ICAM-1 expression by 84.5% ± 1.9%, 78.8% ± 1.2%, 58.9% ± 0.4%, 58.7% ± 0.9%, and 57.4% ± 0.3% correspondingly (Chen et al., 2001). By suppressing the expressions of YAP/TAZ and inflammation-related proteins (NF-κB, TNF-α, and JNK) in the VECs, SAB shields them from inflammatory damage and delays the development of AS (Yang et al., 2020b). This process is strongly linked to the control of the YAP/TAZ/JNK pathway over oxidized LDL (ox-LDL) formation and its anti-inflammatory properties. The suppression of NF-κB-regulated proinflammatory mediators, which can dramatically and dose-dependently downregulate the expressions of ICAM-1, IL-1 β, IL-6, IL-8, and monocyte chemoattractant protein-1 (MCP-1), is also linked to the anti-inflammatory actions of SAB (Xu et al., 2015). Since interferon gamma (IFN-γ) is a major cytokine that causes AS, blocking its effects on the inflammatory responses could be a valuable strategy for preventing AS (Li et al., 2023b). SAB suppresses the Janus kinase (JAK)-signal transducer and activator of transcription (STAT) signaling pathway triggered by IFN-γ, in addition to JAK2 and STAT1 phosphorylation and IP-10 promoter activity. These lower the release of CXC chemokines, lowering T-cell recruitment and slowing the progression of AS (Chen et al., 2011). PCA demonstrates significant anti-inflammatory properties in an IL-1β-induced endothelial cell model of diabetic endothelial dysfunction, primarily through enhancement of the phosphorylation of both endothelial nitric oxide synthase (eNOS) and aktin protein kinase B (Akt) (treatment is dose-dependent with statistical significance at 100 nM). Notably, pharmacological inhibition of Akt phosphorylation resulted in sustained reduction of p-eNOS/eNOS levels and abrogated PCA’s suppressive effects on proinflammatory cytokine production, suggesting that the anti-inflammatory actions of PCA are mediated by the Akt/eNOS signaling pathway (Chook et al., 2023). The above studies suggest that SAA and SAB are potential candidates for anti-inflammatory actions against VECs.

#### 3.1.3 Inhibition of endothelial pyroptosis

The development of AS is tightly linked to VEC pyroptosis, which is a novel form of inflammatory cell programmed death (He et al., 2021; Zhaolin et al., 2019). Furthermore, the role of NLRP3 inflammatory vesicles in VEC pyroptosis has been demonstrated (Lv et al., 2024). Plaque vulnerability and overexpression of NLRP3 inflammasome-related components were closely correlated, indicating that NLRP3 inflammasome activation may encourage plaque instability (Paramel Varghese et al., 2016). DSS mainly prevents VEC juxtaposition at the initial stage of NLRP3 activation. DSS binds to the recombinant chloride intracellular channel protein 4 (CLIC4) and blocks its membrane localization to suppress the release of IL-1β and IL-18, cleavage of pro-caspase-1, and activation of NLRP3 inflammatory vesicles (Zhang et al., 2024b). In contrast, SAA (5, 10, and 25 µM) dose-dependently interacts with pyruvate kinase isozyme typeM2 (PKM2) to prevent Y105 from being phosphorylated and to prevent PKM2 from moving to the nucleus to cause SAA regulation of cellular focal death dependent on PKM2 (Zhu et al., 2022). Salvianolic acids can thus prevent AS-induced VEC pyroptosis.

#### 3.1.4 Inhibition of endothelial oxidative stress

Oxidative stress of the vascular endothelium is a significant precursor to AS (Suschek et al., 2003). One of the major causes of endothelial dysfunction is increase in ROS buildup in the vessel walls (Ray and Shah, 2005). By modulating the SIRT1-eNOS pathway, SAA (100, 200, and 400 mg/L) dramatically enhances cell viability and inhibits the formation of excess nitric oxide (NO) and ROS in response to HG circumstances in a dose-dependent manner (Zhai et al., 2019). SAA also exerts endothelial protective effects by directly reducing malondialdehyde (MDA), advanced glycation end products (AGEs), NOS activity, and endothelial-type NOS protein expression in rats to inhibit oxidative stress (Yang et al., 2011). SAB (25 mg/kg/d) decreases the increased expression of angiotensin I (AT1) receptor, nicotinamide adenine dinucleotide phosphate hydrogen (NADPH) oxidase, nitrotyrosine, NADPH oxidase (NOX)-2, and NOX-4 proteins as well as excess generation of ROS in the vascular walls (Ling et al., 2017). This was shown to restore damaged endothelial function and dramatically reduce AT1 receptor-dependent vascular oxidative stress. The primary sources of ROS in the vascular system are NOX-2 and NOX-4, which are the two main isoforms of NADPH oxidase (Harel et al., 2017). SAB (80 and 160 mg/kg) was shown to lower the oxidative stress levels in a rat model of vascular endothelial dysfunction caused by blood glucose oscillations, as observed through decreased levels of NOX-2 and NOX-4 proteins as well as MDA (5.367 ± 0.89 and 4.467 ± 0.83, respectively) in the aorta (Ren et al., 2016). The bone morphogenetic protein 4 (BMP4)-ROS cycle in db/db mice causes oxidative stress through BMP4 overexpression, contributing to endothelial dysfunction. SAB improves endothelial function by interfering with the BMP4-ROS cycle to suppress p38 MAPK/JNK/caspase-3 activation (Liu et al., 2021b). Consequently, SAA and SAB may be useful natural substances for treating endothelial dysfunction caused by oxidative stress injury in vascular disorders.

#### 3.1.5 Inhibition of endothelial apoptosis

AS is triggered and promoted by endothelial cell apoptosis (Paone et al., 2019). Thus, one of the most successful preventative and therapeutic approaches for AS involves the use of substances with antiapoptotic properties (Duan et al., 2021). As previously stated, SAB has antioxidant and anti-inflammatory properties that guard against damage to VECs caused by inflammation, oxidative stress, and others. In apoptosis caused by these harmful conditions, SAB has also shown potent antiapoptotic actions. Using the phosphatidylinositol 3-kinase (PI3K)/Akt/rapidly accelerated fibrosarcoma kinase (Raf)/MEK/ERK pathway, hydrogen peroxide (H_2_O_2_) causes VEC death while SAB (20 µM) shields rCMECs from the corresponding processes (Liu et al., 2007). SAB also inhibits oxidative stress, p53, and caspase-3 production, which results in antiapoptotic actions in ox-LDL-induced cellular damage (Chen et al., 2017). SAB (60 μg/mL) promotes antiapoptotic effects by increasing antioxidant enzyme activity, inhibiting inflammatory factor secretion, and restoring balanced expressions of proapoptotic and antiapoptotic proteins via the p38MAPK/NF-κB and PI3K/Akt/NF-κB pathways mediated by lectin-like ox-LDL receptor 1 (LOX-1), NOX-4, and ERα receptors (Yang et al., 2018). Here, ox-LDL triggers the intrinsic apoptotic pathway of VECs implicated in mitochondrial autophagy and apoptosis when the glu levels are elevated. Conversely, SAB (1, 10, 50, and 100 µM) dose-dependently protects VECs harmed by ox-LDL in HG circumstances and suppresses the rho-associated coiled-coil containing protein kinase 1 (ROCK1)-mediated apoptotic pathway to prevent apoptotic cell death and mitochondrial breakage (Ko et al., 2020). SAA (100, 200, and 400 mg/L) also prevents apoptosis through elevated glu by upregulating B-cell lymphoma-2 (Bcl-2), downregulating Bcl-2 associated X (Bax), and blocking caspase-3 and caspase-9 activities in a dose-dependent manner (Zhai et al., 2019). In addition to the cellular milieu of elevated glucose, variations in blood glucose levels can induce apoptosis and vascular endothelial dysfunction. In rats with blood glucose fluctuations, SAB was shown to significantly enhance endothelial function; this was achieved by inhibiting oxidative stress, upregulating Bcl-2 proteins, and considerably downregulating Bax proteins, thereby preventing apoptosis of VECs (Ren et al., 2016; Xiang et al., 2022). In conclusion, salvianolic acids prevent apoptosis of VECs and endothelial dysfunction caused by several sources.

#### 3.1.6 Inhibition of endothelial autophagy

The development and instability of AS plaques are facilitated by impaired autophagy that contributes to inflammation and apoptosis (Martinet and De Meyer, 2009; Sergin and Razani, 2014). The creation and rupture of AS plaques is facilitated by oxidatively damaged lipoproteins, inflammatory agents, and metabolites that trigger cellular autophagy, particularly in VECs (Siddiqui et al., 2015). SAB inhibits autophagy in HUVECs by controlling the expressions of microtubule-associated protein light chain 3 (LC3), Beclin1, and P62 via miR-19a/SIRT1 (Guo et al., 2021). SAB at 40 and 80 µM dramatically elevated the levels of Bcl-2, LC3, autophagy-related protein 5 (Atg5), and Beclin1 in response to glucose restriction, while 80 µM of SAB decreased the expressions of Bax, lactate dehydrogenase (LDH), MDA, and superoxide dismutase (SOD) (Chen et al., 2023). However, these protective effects of SAB were lost when Atg5 expression was suppressed, implying that SAB may protect the vasculature by controlling the autophagy of VECs.

#### 3.1.7 Improvement of endothelial mitochondrial dysfunction

Mitochondria are the prominent organelles of energy production whose dysfunction is an essential pathological basis for endothelial dysfunction, which is closely related to the occurrence and development of cardiovascular diseases (Karnewar et al., 2022). Mitochondrial autophagy plays a crucial role in the development of AS; it directly eliminates damaged and defective mitochondria to preserve regular mitochondrial quantity and quality, which are necessary for organisms to maintain healthy mitochondrial homeostasis (Chen et al., 2022d; Pickles et al., 2018; Yang et al., 2020a). Under oxidative stress or inflammatory stimulation conditions, VECs undergo mitochondrial dysfunction characterized by decreased membrane potential and impaired electron transport chain activity to trigger protective autophagy, which eliminates the damaged mitochondria and maintains cellular homeostasis (Zong et al., 2015). However, persistent pathological stimuli can lead to autophagic flux impairment owing to lysosomal system overload or defective lysosomal acidification, resulting in failed degradation of dysfunctional mitochondria. This accumulation of abnormal mitochondria ultimately drives the transition from cytoprotective autophagy to apoptotic cell death in VECs. SAA stimulates mitochondrial production in VECs by activating the adenosine 5′-monophosphate (AMP)-activated protein kinase (AMPK)-mediated peroxisome proliferator-activated receptor gamma coactivator 1-alpha (PGC-1α)/transcription factor a mitochondrial (TFAM) signaling pathway (Wang et al., 2022), which markedly increases AMPK and ACC phosphorylation. SAB dramatically reduces Parkin and Pink1 expressions, downregulates mitochondrial autophagy, and increases mitochondrial activity in HG-induced HUVECs (Xiang et al., 2022); therefore, salvianolic acids may be beneficial natural substances for treating vascular diseases by addressing mitochondrial dysfunction.

### 3.2 Effects on EPCs

**FIGURE 5:**
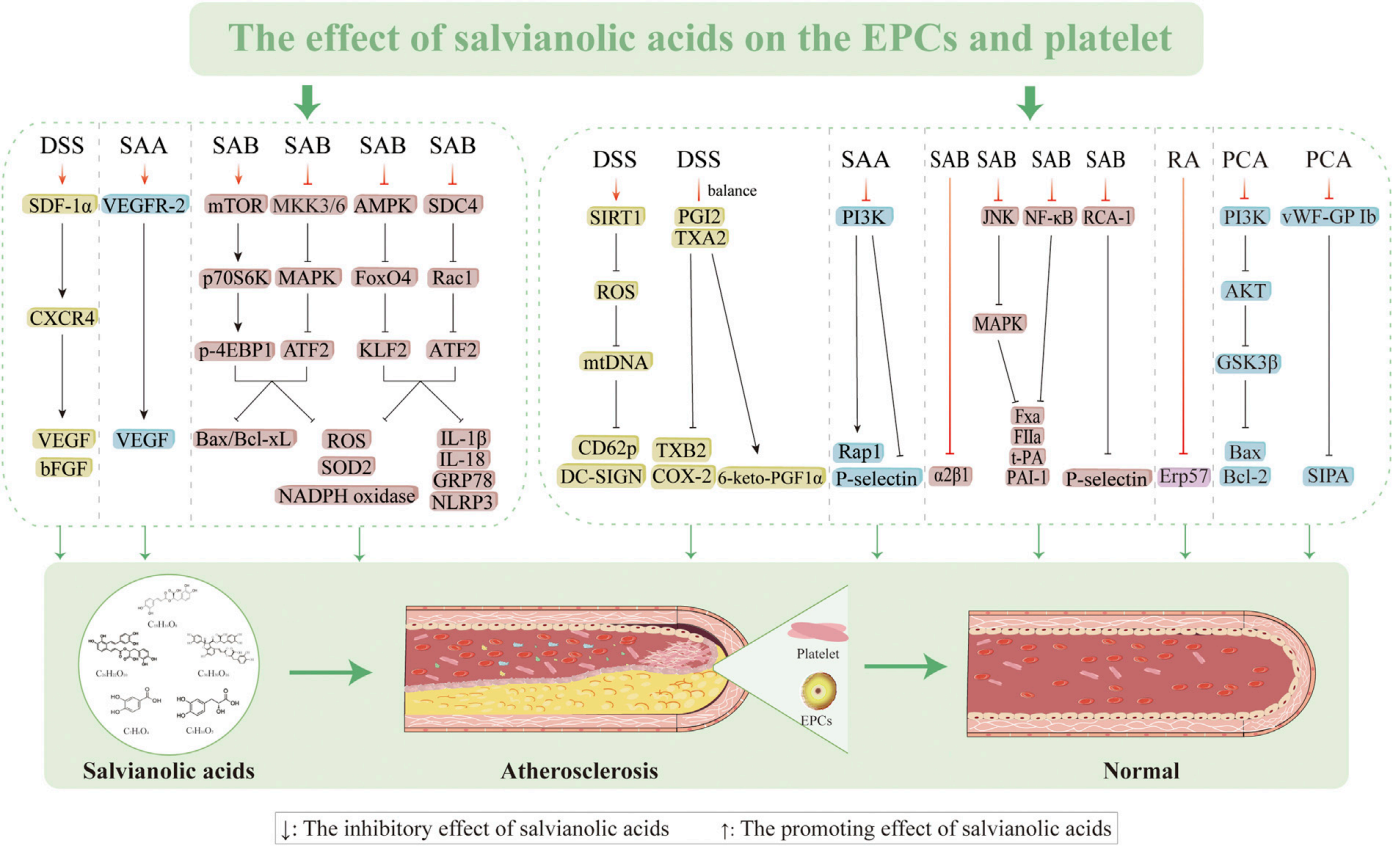
Effects of salvianolic acids on vascular smooth muscle cells.

Proliferation, migration, and differentiation of EPCs into mature VECs in the peripheral blood circulation is primarily responsible for the repair process following VEC injury and limited repair induced by the extension of nearby mature VECs (Endtmann et al., 2011). The quantity, migration, proliferation, and cytokine production of circulating EPCs influence the extent to which well-damaged arteries undergo endothelial repair. This information can be used to evaluate endothelial function and forecast the progression of AS. Therefore, there is a critical necessity to determine methods to increase the quantity and functionality of EPCs for preventing and treating AS. DSS (60 mg/kg/d) increases the survivability of EPCs, prevents apoptosis, and improves EPC capacity to migrate and create lumens by encouraging stromal cell-derived factor 1 alpha (SDF-1α) and CXC motif chemokine receptor 4 (CXCR4) expressions as well as AKT phosphorylation. AMD3100 (a CXCR4 antagonist) and LY294002 (a PI3K antagonist) have been reported to partially inhibit this ability (Yin et al., 2017). However, SAA (2.5, 5.0, and 10 mg/kg/d) can enhance neovascularization by upregulating VEGF, VEGFR-2, and MMP-9 expressions while preserving the quantity and functionality of EPCs (Li et al., 2014). SAB triggers the mechanistic target of the rapamycin (mTOR)/p70 ribosomal protein S6 kinase (p70S6K)/eukaryotic translation initiation factor 4E-binding protein 1 (4EBP1) pathway, thereby inhibiting the expressions of mitogen-activated protein kinase kinase (MKK) 3/6-p38MAPK-activating transcription factor 2 (ATF2) and ERK1/2 while increasing EPC migration, improving tube formation, and preventing cellular damage caused by H_2_O_2_ (Tang et al., 2014). The present study reveals the molecular processes in the oxidative signaling cascade involved in SAB; we identify potential targets for intervention to prevent endothelial-damage-mediated vascular injury along with applications concerning endothelial-damage-mediated vascular disease and downregulated ROS levels (162.0% ± 9.9% and 136.7% ± 13.9, respectively). However, endoplasmic reticulum stress (ERS) causes BM-EPCs to produce NLRP3 and release mitochondrial ROS. Meanwhile, SAB prevents endothelial damage linked to ERS by controlling the adhesion proteoglycan-4/Rac1/ATF2 and AMPK/FOXO4/Krüppel-like factor (KLF)2 pathways, thereby preventing NLRP3 inflammasome-mediated cellular pyroptosis (Tang et al., 2022). Thus, salvianolic acids mitigate vascular endothelial damage, improve EPC mobilization, and restore EPC functions by efficiently inhibiting ERS, oxidative stress, and apoptosis while restoring their migratory capacity (Figure 5).

### 3.3 Inhibition of VSMC proliferation and migration

**FIGURE 6:**
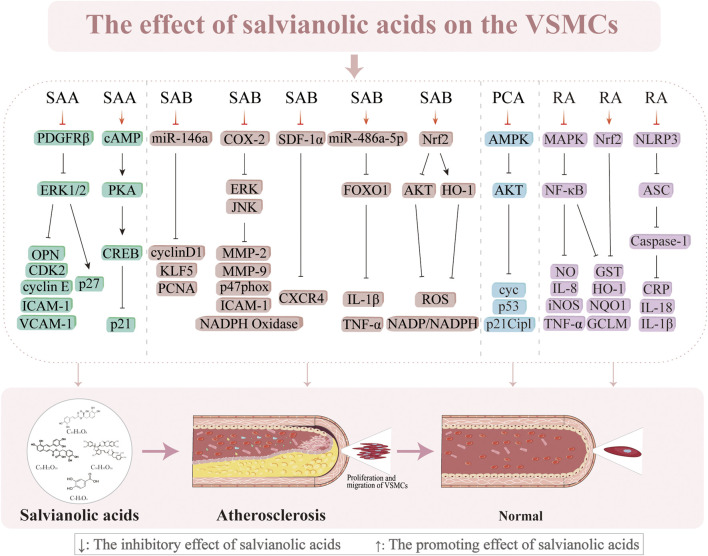
Effects of salvianolic acids on endothelial progenitor cells and platelets.

VSMC proliferation and migration are essential factors in the pathogenesis of AS (Basatemur et al., 2019). VSMCs multiply, move to the subendothelial layer of the vasculature, and contribute to the formation of plaques when the endothelial cells are injured (Wang et al., 2015a). The progression of AS is significantly influenced by this proliferation and migration, which occur early in the process. Sun et al. (2012) discovered that SAA (0.01–0.1 µM) blocks the platelet-derived growth factor receptor (PDGFR) β-ERK1/2 cascade to prevent PDGF-BB from causing VSMC migration and proliferation in a concentration-dependent manner. However, the influence of SAA on VSMC proliferation is not limited to stopping the cell cycle in the G0/G1 phase; it is also intimately linked to the cyclic adenosine monophosphate (cAMP)/protein kinase a (PKA)/camp response element-binding protein (CREB) signaling cascade, which inhibits endothelial proliferation and increases p21/Cip1 (Sun et al., 2012; Sun et al., 2016b). However, SAB (5 μmol/L) suppresses the growth of VSMCs produced by Ang II and endothelial hyperplasia caused by carotid artery ligation *in vitro* by downregulating miR-146a and the positive cell cycle regulators KLF5 and cyclin D1 (Zhao et al., 2019). In addition, SAB (10 µM) significantly decreases the intima/media area ratio (0.010% ± 0.009%) by blocking lipopolysaccharide (LPS)-induced expression of cyclooxygenase-2 (COX-2) from 3.8 ± 0.3 to 1.5 ± 0.2, inhibiting ERK and JNK phosphorylation, attenuating prostaglandin E2 production, and increasing NADPH oxidase activity (Chen et al., 2006). SAB also successfully improves the suppression of FOXO1 activity and increases the expression of miR-486a-5p to reduce inflammation caused by HG in VSMCs (Zhang et al., 2024a). SDF-1α has an important regulatory role in VSMC migration and proliferation during neointimal development (Li et al., 2012b). SAB successfully suppresses chemokine (CXC motif) receptor 4 (CXCR4) expression and prevents SDF-1α/CXCR4-induced cell migration and proliferation (Pan et al., 2012). This implies that SAB might prevent the growth of neoplastic endothelial hyperplasia by acting as a CXCR4 receptor antagonist. According to numerous studies, MMPs, specifically MMP-2 and MMP-9, facilitate VSMC migration from the mid-membrane to the endothelium, resulting in neointimal hyperplasia (Brauninger et al., 2023; Dai et al., 2019; Sun et al., 2016a). SAB (5, 10, and 20 µM) inhibits MMP-2 and MMP-9 activities as well as downregulates JNK and ERK phosphorylation in a concentration-dependent manner to decrease LPS-induced migration of VSMCs (Lin et al., 2007). The antioxidant protein heme oxygenase-1 (HO-1) prevents the formation of AS and shields vascular cells against neointimal hyperplasia after vascular injury (Juan et al., 2001; Tulis et al., 2001). SAB prevents PDGF-induced VSMC migration and proliferation by scavenging ROS and upregulating HO-1 expression (Lee et al., 2014). Research has demonstrated that RA exerts multiple protective effects on VSMCs through distinct molecular mechanisms, such as suppression of PDGF-BB-induced VSMC proliferation, migration, and phenotypic switching via activation of the nuclear factor erythroid 2-related factor 2 (Nrf2)/antioxidant response element (ARE) pathway (Chen et al., 2022a); RA mitigates nicotine-induced vascular inflammation and retards AS progression by inhibiting the ROS-NLRP3-CRP axis as well as attenuates LPS-induced inflammatory responses in VSMCs through blockade of the MAPK/NF-κB pathway (Chen et al., 2022b). Furthermore, PCA has been shown to inhibit A7r5 cell proliferation through AMPK pathway activation mediated by downregulation of fatty acid synthase (FAS) expression, suppression of AKT phosphorylation and S-phase kinase-associated protein 2 (Skp2) protein synthesis, and induction of cell cycle arrest at the G0/G1 phase via coordinated activation of both the p53/p21Cip1 and Skp2 pathways (Lin et al., 2015). Subsequent research has shown that HO-1 knockdown reduces the capacity of SAB to control atherogenic processes in VSMCs induced by PDGF. These works demonstrate the pivotal functions of HO-1 and the contributions of its activation to the anti-atherosclerotic actions of SAB. Salvianolic acids may thus work against AS through various mechanisms, including antiproliferative and antimigratory actions on VSMCs (Figure 6).

### 3.4 Regulation of macrophages in AS

The presence of a mass infiltration of macrophages is one of the pathological underpinnings for the development of AS (Wilson, 2022). One of the factors contributing to plaque development in AS is macrophages, which are engaged in intraplaque neovascularization, fibrous cap, lipid necrotic core, and plaque inflammation (Martinet and De Meyer, 2009). The uptake of ox-LDL by macrophages initiates a pathological cascade characterized by cholesterol crystal (CHC) deposition, which triggers inflammasome activation and subsequent release of proinflammatory cytokines, including IL-1β, thereby promoting macrophage polarization toward the proinflammatory M1 phenotype (Luo et al., 2025). This M1 polarization establishes a self-amplifying inflammatory loop through ROS generation to exacerbate cellular apoptosis. When the apoptotic macrophages fail to undergo timely efferocytosis by the neighboring cells, they progress to secondary necrosis and release proinflammatory contents that contribute to necrotic core formation. In advanced stages of AS, persistent lipid accumulation and the sustained inflammatory microenvironment suppress anti-inflammatory M2 polarization, impairing both efferocytosis and cholesterol efflux mechanisms. These pathological alterations lead to progressive lipid accumulation, necrotic core expansion, and fibrous cap thinning to ultimately promote plaque destabilization and accelerate atherosclerotic progression (Farahi et al., 2021).

#### 3.4.1 Suppression of macrophage inflammation

Inflammation is a hallmark of the pathophysiological processes involved in AS, and macrophages are crucial in these events. The production of foam cells from macrophages in the subendothelial region, which coincides with macrophage proliferation during AS plaque formation, is a characteristic of early AS (Emanuel et al., 2014; Lv et al., 2014; Shen et al., 2023). Furthermore, compared to stable plaques, susceptible plaques in AS exhibit more significant macrophage infiltration (Seneviratne et al., 2013). Therefore, inflammatory reactions mediated by macrophages play essential roles in the formation of AS. Reduction of the inflammatory responses, prevention of plaque rupture, and improvement of AS progression depend on inhibiting macrophage infiltration and multiplication (Tang et al., 2015). AS development is tightly linked to the classical NF-κB signaling pathway, which is mediated by the inhibitor of nuclear factor kappa-B kinase (IKKβ) subunit (Gwon et al., 2020). DSS demonstrates strong anti-inflammatory effects in macrophages through the IKKβ/NF-κB pathway. These effects have been primarily observed in the inhibition of *Ltf*, *Ccr7*, *IFN-γ*, and *Cxcl9* expressions; reductions of arterial plaque development and areas in mice; and improvement of the stability of susceptible plaques (Ye et al., 2020; Zeng et al., 2023). LPS stimulates the release of proinflammatory factors involved in inflammatory reactions, translocation of NF-κB to the nucleus, and phenotypic shift of macrophages to the M1 phenotype (Bosca et al., 2005; Herrera-Velit et al., 1997; Jeong et al., 2005; Moller et al., 2012; Ni Gabhann et al., 2014; Shapira et al., 1996). By blocking IKKα/β activation via the p38-HO-1 pathway through blocking of IKKα/β phosphorylation and NF-κB translocation to the nucleus, SAA might decrease the transcriptional activation of NF-κB; this suggests that SAA has anti-inflammatory effects on LPS-stimulated RAW 264.7 cells. The toll-like receptor 4 (TLR4) is an essential receptor of LPS that promotes inflammation (Baeuerle, 1998). Induction of THP-1 cells with LPS (1 μg/mL) for 24 h was shown to significantly upregulate proinflammatory cytokines, with the expression levels of IL-1β, IL-6, and TNF-α reaching 60.4 ± 5.5, 109.1 ± 19.5, and 1,297.29 ± 94.09, respectively, demonstrating successful induction of M1 macrophage polarization. SAB (0.72, 3.60, and 17.95 μg/mL) exerts anti-inflammatory effects through the TLR4/NF-κB signaling pathway, significantly reducing the levels of LPS-induced TLR4, p-IκBα, and p-p65 proteins as well as preventing nuclear translocation of NF-κB and degradation of IκBα (Liu et al., 2018a). Furthermore, SAB inhibition of LPS-induced macrophage inflammation has been linked to increased arginase activity, decreased NO generation, and increased expression of the cytoprotective protein HO-1 (Joe et al., 2012). RAW264.7 cells induced with ox-LDL (100 μg/mL) or LPS (100 μg/mL) for 24 h showed significantly increased secretion of proinflammatory cytokines, including IL-1β, IL-6, TNF-α, and iNOS, confirming successful polarization toward the M1 macrophage phenotype. By preventing activation of the MAPKs/NF-κB pathway, reducing lipid buildup, and reducing the expressions of inflammatory components in the aorta and RAW264.7 cells, SAB (25 mg/kg) has been shown to exert anti-atherosclerotic effects in high-fat-diet-induced LDLR^−/−^mice, ox-LDL, or LPS-induced RAW264.7 cells (1.25, 2.5, and 5 μg/mL) (Zhang et al., 2022). Therefore, one of the key biological targets of salvianolic acids for anti-atherosclerotic effects is the inhibitory action on macrophage inflammation caused by various stressors.

#### 3.4.2 Inhibition of macrophage polarization

The quantity and polarization state of the invading macrophages determine the size and stability of the plaques (Xia et al., 2020). By lowering the amount of the membrane receptor protein Mincle and phosphorylating the downstream effector molecule Syk, SAB prevents macrophage M1 phenotype (proinflammatory) polarization, highlighting that Mincle is a target protein for the anti-inflammatory actions of SAB (Li et al., 2021d). One of the methods by which SAB controls macrophage polarization to provide anti-inflammatory effects is promoting the growth of anti-inflammatory (M2) phenotype macrophages while simultaneously suppressing the number of M1 phenotype macrophages. Niu et al. (2022) found that SAB (15 mg/kg/d) supports blood perfusion by increasing the quantity of M2 phenotype macrophages via the SIRT1/PI3K/AKT pathway. Following 24 h of stimulation with LPS (100 ng/mL) and IFN-γ (2.5 ng/mL), RAW264.7 cells exhibited morphological changes from round to oval or spindle shapes and increased expressions of M1 markers (iNOS, TNF-α, and IL-6), thereby confirming successful M1 polarization. Treatment with SAB (100, 150, and 200 µM) significantly downregulated M1-associated proinflammatory factors while upregulating M2 markers (Arg-1 and IL-10), indicating SAB-mediated M1-to-M2 phenotype switching. This phenotypic transformation was found to be autophagy-dependent, as evidenced by the reversal of LC3-II, Beclin1, and p62 alterations upon 24 h of treatment with the autophagy inhibitor 3-MA (5 mM). Further mechanistic studies using the protein kinase C (PKC) agonist PMA (100 nM/mL) revealed the involvement of NF-κB pathway activation and AKT/mTOR inhibition in SAB-induced autophagy. These findings collectively demonstrate that SAB promotes macrophage phenotype switching through autophagy modulation via NF-κB activation and AKT/mTOR pathway inhibition (Zou et al., 2022). Zhao et al. (2020) demonstrated that LPS (100 ng/mL) stimulation of BMDMs for 24 h significantly upregulated mRNA expressions of the M1 polarization markers (TNF-α, IL-6, iNOS, and CCL2), confirming successful M1 polarization. Conversely, SAB treatment (5, 10, and 20 µM) exerted concentration-dependent effects, including inhibition of mTORC1-mediated glycolysis, downregulation of M1 marker expressions (IL-6, iNOS, CCL2, and TNF-α), and upregulation of M2-associated genes (*Arg1*, *Clec10a*, and *Mrc*); these coordinated effects promote macrophage switching from proinflammatory M1 to anti-inflammatory M2 phenotype polarization (Zhao et al., 2020). PCA exerts dual regulatory effects on macrophage polarization by suppressing M1 polarization through inhibition of the PI3K-AKT/NF-κB signaling pathway and promoting M2 polarization by activating both STAT6 and peroxisome proliferator-activated receptor (PPAR)γ pathways. This coordinated modulation of the M1–M2 phenotypic transition in both J774 cells and mouse bone-marrow-derived macrophages contributes to its therapeutic efficacy in alleviating AS progression (Liu et al., 2019). These findings provide a better theoretical basis for further research and development of salvianolic acids as therapeutic agents for restoring macrophage polarization and treating AS.

#### 3.4.3 Inhibition of macrophage apoptosis

**FIGURE 7:**
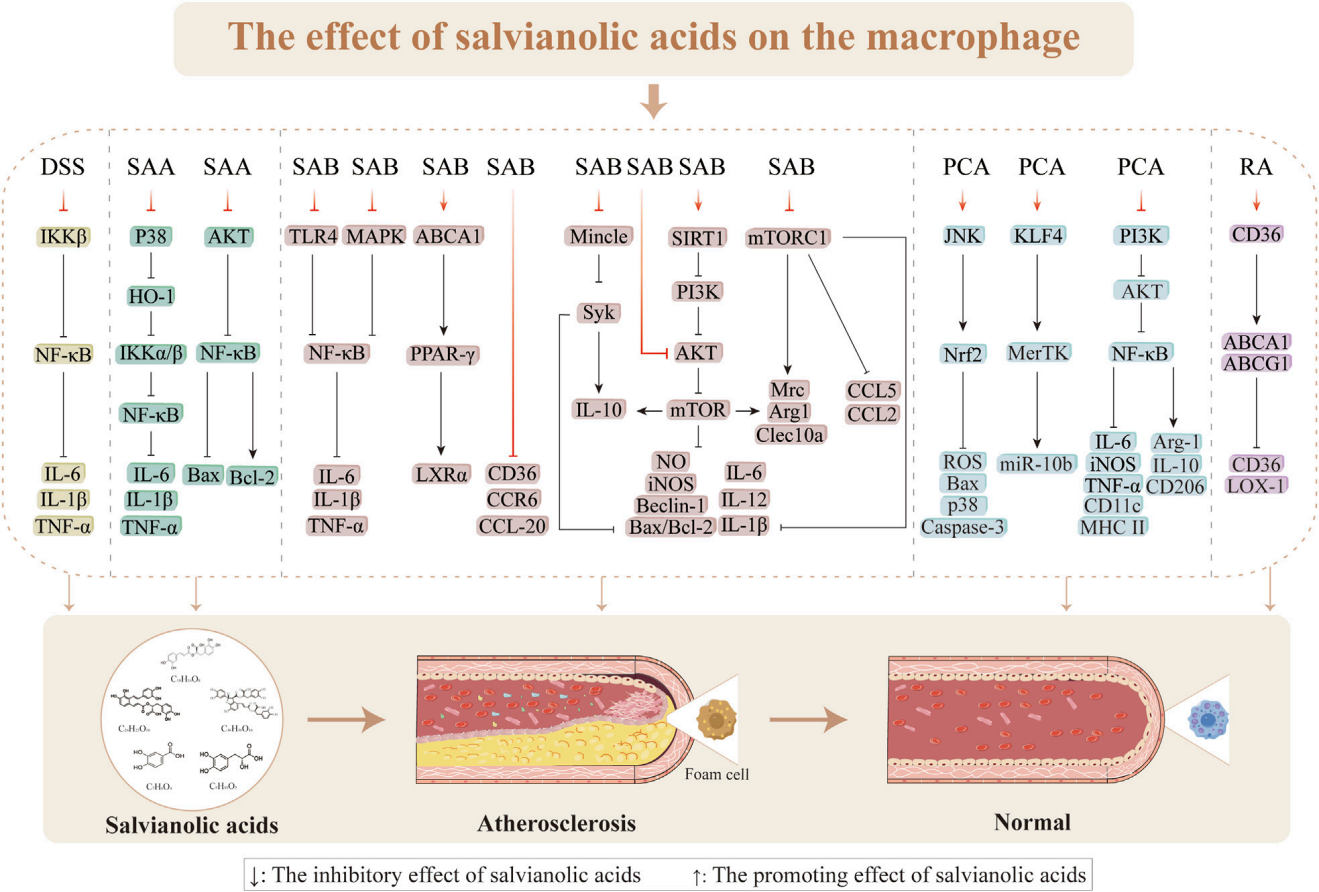
Effects of salvianolic acids on macrophages.

One of the treatment strategies for advanced AS is decreasing macrophage apoptosis while preserving phagocytosis as this process exacerbates plaque development (Simion et al., 2020). SAA has similar effects to PI3K inhibitors in that it counteracts Ang-II-induced macrophage apoptosis and oxidative stress (Li et al., 2016). These effects are demonstrated by the increase in TUNEL-positive cells, upregulation of SOD and Bax expressions, and decreases in Bcl-2, MDA, p-NF-κB, p-AKT 473, and p-AKT 308 expressions. Here, autophagy is controlled by the inhibitory AKT/mTOR signaling pathway (Chang et al., 2013). According to research, the buildup of CHCs and subsequent inflammatory responses are intimately linked to the instability of AS plaques (Duewell et al., 2010; Whiting and Watts, 1983). Sun et al. (2021) showed that SAB reduces apoptosis in RAW264.7 cells and improves autophagy dysfunction caused by CHC by modulating the AKT/mTOR signaling pathway. PCA (25 µM) promotes nuclear translocation of Nrf2 and suppresses ROS overproduction during the initial phase of oxidative stress via JNK pathway activation; it also mediates JNK/Nrf2 signaling-dependent regulation of the endogenous antioxidant defense system. This coordinated action plays a crucial role in the cytoprotective properties of PCA against oxidative-stress-induced apoptosis in macrophages (Vari et al., 2015) (Figure 7).

#### 3.4.4 Improvement of macrophage lipid metabolism

One of the leading causes of AS lesion progression is disturbed macrophage lipid metabolism along with foam cell production (Li et al., 2021c). AS development can be slowed by boosting cholesterol export because the accumulated cholesterol encourages macrophages to convert into foam cells (Moore et al., 2013). SAB stimulates the ox-LDL-activated THP-1 cells to increase apoA-1 and HDL-mediated cholesterol efflux through the ABCA1/PPAR-g/LXRα pathway (Yue et al., 2015). Although CD36 overexpression has been shown to prevent ischemia–reperfusion injury, CD36 deficiency can reduces the progression of AS (Shu et al., 2022). Wang et al. (2010b) suggested SAB (1, 10, and 100 µM) as one of the potential natural compounds for CD36 antagonism (where CD36 levels were reduced by 11.2%, 48.9%, and 78.3%, respectively); here, SAB had a higher binding affinity, indicating that it was a more potent CD36 antagonist than hexarelin (kinetics by association: 264.3 M^−1^s^−1^ vs. 240.1 M^−1^s^−1^; dissociation constant: 9.89 × 10^−4^ s^−1^ vs. 5.48 × 10^−3^ s^−1^; affinity: 3.74 μM vs. 22.8 μM) that binds directly to CD36 to reduce lipid uptake (17.65%). SAB was found to exert this effect in a CD36-dependent manner after knockdown or overexpression of CD36 in macrophages (Bao et al., 2012; Wang et al., 2010b); this implies that SAB has a strong and targeted inhibitory effect on the CD36 pathway. One of the chemokines essential for the development of AS that accumulates in the plaques is CC chemokine ligand-20 (CCL-20)/macrophage inflammatory protein-3α (Schutyser et al., 2003). Reduced lipid accumulation and decreased AS plaque extension were the outcomes of considerable inhibition of CCL-20 production and CCR6 expression by SAA in TNF-α-stimulated RAW264.7 cells (Zhang et al., 2014). Additionally, SAA was shown to suppress p-p38 through the MAPK pathway. RA enhances the cholesterol efflux capacity in macrophages by upregulating ATP-binding cassette transporter (ABC)A1 and ABCG1 expressions. Mechanistic studies reveal that RA differentially regulates ABCG1 expression via multiple signaling pathways, including JAK2/STAT3 activation, JNK modulation, and PKC-mediated regulation of the ERK1/2/p38 MAPK cascade (Nyandwi et al., 2021). According to the above reports, salvianolic acids are beneficial for enhancing lipid metabolism in macrophages and controlling AS thereof.

#### 3.4.5 Modulation of macrophage cytostasis

Macrophages play a crucial role in tissue homeostasis through efferocytosis, which is the process of apoptotic cell clearance. However, this essential function is significantly impaired in advanced atherosclerotic plaques, contributing to sustained inflammation and necrotic core expansion (Doran et al., 2020; Morioka et al., 2019; Yurdagul et al., 2017). Mechanistic studies have shown that PCA enhances macrophage efferocytic capacity through a novel miR-10b-mediated pathway and that PCA promotes microRNA-10b secretion into the extracellular vesicles, thereby reducing intracellular miR-10b levels; this reduction derepresses KLF4, which is a key transcriptional regulator, and subsequently upregulates MerTK receptor expression to enhance efferocytic efficiency (Li et al., 2023a). Oral PCA administration has been reported to significantly improve macrophage efferocytosis in atherosclerotic plaques via the miR-10b-KLF4-MerTK axis, resulting in reduced apoptotic cell accumulation and attenuated inflammatory responses *in vivo* (Li et al., 2023a). These findings not only establish the therapeutic potential of PCA through activation of the KLF4-MerTK pathway but also provide a novel strategy for AS management by targeting the efferocytic mechanisms, thereby highlighting the therapeutic potential of modulating apoptotic cell clearance in cardiovascular pathologies.

### 3.5 Effects on monocyte-derived dendritic cells

Foam cell formation is crucial for the development of AS and is mediated by monocyte-derived dendritic cells using exophagic breakdown of aggregated LDLs (Haka et al., 2015). By inhibiting the TLR4/MyD88-mediated p38-MAPK pathway as well as upregulating PPARγ nuclear translocation and transcription through the PPARγ signaling cascade, SAB efficiently prevents ox-LDL-induced maturation of h-monDC to consequently reduce the immunological responses and cellular inflammation (Sun et al., 2011). PCA also demonstrates significant anti-atherogenic effects by suppressing monocyte adhesion to TNF-R-activated endothelial cells, inhibiting NF-κB pathway activation, and attenuating early atherosclerotic lesion formation *in vitro* (Wang et al., 2010a).

### 3.6 Inhibition of platelet activation and antithrombosis

AS develops as a result of alterations in the vascular structure and functions caused by various mechanical and chemical factors that damage the VECs. This allows infiltration of the inflammatory cells, adhesion and aggregation of platelets, and deposition of fibrin to form thrombi (Massberg et al., 2010; Pan et al., 2009). Therefore, prevention of platelet aggregation is crucial for the management of cerebrovascular and cardiovascular disorders (Davi and Patrono, 2007).

DSS exerts potent antiplatelet effects through SIRT1/ROS/mtDNA pathway modulation, demonstrating a concentration-dependent inhibition (1–500 µM) of platelet aggregation induced by multiple agonists, including U46619 (1 µM), TRAP-6 (2 µM), and adenosine diphosphate (ADP; 10 µM). This unique mechanism provides effective antithrombotic protection without compromising hemostatic balance, thereby minimizing bleeding risk; however, the SIRT1 inhibitor EX-527 (10 µM) reverses this effect, indicating that DSS suppresses ROS generation and prevents platelet activation by upregulating SIRT1 expression (Xue et al., 2022). The capacity of DSS to alter blood rheology without compromising the coagulation system is linked to its antithrombotic action. In a comprehensive preclinical evaluation, male SD rats and ICR mice received oral administration of DSS (15, 30, and 60 mg/kg) or aspirin (20 mg/kg) for 7 d. Pharmacological assessments were then conducted using both *in vitro* platelet aggregation assays and an *in vivo* arteriovenous shunt thrombosis model complemented by a venous thrombosis model. The results showed that DSS treatment significantly improved the hemorheological parameters, as evidenced by the lower blood and plasma viscosities, while inhibiting platelet aggregation caused by arachidonic acid (AA) and ADP. This can be explained by the ability of DSS to precisely suppress COX-2 while restoring the equilibrium between prostacyclin (PGI2) and thromboxane A2 (TXA2). Additionally, SAA has good antithrombotic activity, as evidenced by its dose-dependent inhibition of thrombus growth in the arteriovenous shunt model, activation of adenylate cyclase to increase cAMP levels for inhibiting platelet aggregation and release, and improvement of erythrocyte pressure and whole blood viscosity as two indicators of blood rheology (Fan et al., 2010; Yu et al., 2014). Huang et al. (2010) discovered that SAA primarily inhibits the PI3K pathway to influence platelet activation and exert antithrombotic effects. SAA specifically decreases platelet–fibrinogen binding ability and ADP-induced P-selectin expression on the platelet surface, further preventing ADP-mediated platelet–leukocyte aggregation (Yuan et al., 2017). The antithrombotic effectiveness of SAA is further enhanced by its ability to prevent platelet spreading on the fibrinogen matrix efficiently. SAB inhibits platelet adhesion to collagen in a dose-dependent manner by blocking α2β1-collagen interactions, as demonstrated by reduced platelet deposition under physiological shear rates, decreased α2β1 antibody binding in flow cytometry, and inhibited soluble α2β1-collagen binding in solid-phase assays (Wu et al., 2008). SAB interacts with P2Y1 and P2Y12 platelets to modestly reduce adhesion and aggregation (Liu et al., 2014; Liu et al., 2018b).

One of the key targets in the development of antithrombotic medications is the platelet surface protease-activated receptor, which is triggered by thrombin and is a potent agonist of platelet activation. These receptors are essential for platelet activation, aggregation, and blood coagulation (Coppens et al., 2012; Coughlin, 2000; Han et al., 2021). Neves et al. (2024) employed a comprehensive approach combining platelet aggregation assays, coagulation tests, perfusion chamber experiments, and *in vivo* microscopy; using intrinsic fluorescence spectrophotometry and isothermal titration calorimetry, they characterized the thrombin inhibition kinetics and binding affinity of SAB. Their groundbreaking work identified SAB as the first direct plant-derived inhibitor of thrombin, demonstrating dual antithrombotic mechanisms through both thrombin-dependent (active site blockade) and thrombin-independent pathways (Neves et al., 2024). Salvianolic acids have shown great promise in antiplatelet and antithrombotic therapies. Inflammation is an essential factor in thrombosis that is closely related to the coagulation and fibrinolytic systems (Lopes-Bezerra and Filler, 2003; Wei et al., 2022). For example, TNF-α can accumulate in the relevant thrombosis and coagulation pathways, and its overexpression can increase the risk of thrombosis. SAB inhibits TNF-α-induced increases in PAI-1 and TF secretion levels as well as the production of FⅩa and FIIa through the NF-κB/JNK/P38MAPK pathway (Zheng et al., 2021). Platelet apoptosis regulation has emerged as a promising therapeutic strategy for preventing atherosclerotic thrombosis. PCA exerts protective effects by scavenging ROS and preventing H_2_O_2_-induced downregulation of the PI3K/AKT/glycogen synthase kinase 3β (GSK3β) signaling axis, thereby mitigating oxidative-stress-induced platelet apoptosis (Ya et al., 2021). Furthermore, PCA demonstrates significant antiplatelet activity against shear-stress-induced platelet aggregation (SIPA), which is a critical factor in arterial disease progression, by inhibiting the binding of von Willebrand factor to glycoprotein Ib as the primary event initiating SIPA (Kim et al., 2012). Additionally, RA exhibits platelet aggregation inhibition via modulation of ERp57, a protein disulfide isomerase family member, suggesting an alternative mechanism for platelet function regulation (Zou et al., 2018). The above studies demonstrated that salvianolic acids are promising candidates in antiplatelet and antithrombotic therapies (Figure 5).

### 3.7 Regulation of vascular tone

Vasodilatory dysfunction increases the risk of AS while accelerating the breakdown of smooth muscle and endothelial cells (Chen et al., 2024). Rat coronary arteries were shown to be dilated by DSS, with an IC50 value of 71.5 ± 11.0 μg/mL and effective dose ranging from 0.02 to 0.35 mg/mL (Lam et al., 2007). Inhibiting the L-type voltage-dependent Ca^2+^ channels in VSMCs and, to a lesser extent, activating K^+^ channels are the primary mechanisms by which vasodilatory effects are produced (Lam et al., 2007). SAA has a protective effect on impaired vascular reactivity; SAA treatment was shown to restore impaired endothelium-dependent diastolic function in rats by reversing the oxidative stress caused by hyperglycemia as well as improving the aortic ring contractility (Wang et al., 2009). Furthermore, by preventing Ang-II and ET-1 from overactivating the vascular smooth muscle L-type calcium channels, SAA preserves the relaxation function of vascular smooth muscle (Lin et al., 2022). SAA also stimulates F-actin bundling, controls the rearrangement of the actin cytoskeleton, and stabilizes the translation-actin complex, all of which improve coronary artery contractility and blood flow (Zhong et al., 2018). SAB reduces aortic ring relaxation and HUVEC alignment in the direction of shear stress by efficiently blocking mechanical-stimulus-induced Ca^2+^ currents through the Yoda1-induced Piezo1 channels and antagonizing ion flow through these channels (Pan et al., 2022). However, the effective dose of SAB differed between studies. For instance, in a 5-hydroxytryptamine (5-HT)-preconditioned preparation, approximately 0.72 mg/mL of SAB eliminated CaCl_2_-induced vasoconstriction; however, in a KCl-preconditioned preparation, 1.44 mg/mL of SAB resulted in 90% inhibition (Lam et al., 2006). Briefly, salvianolic acids can restore the endothelial-dependent stretch and smooth muscle relaxation functions of blood vessels by modulating vascular mechanotransduction.

## 4 Safety evaluations and clinical evidence for salvianolic acids

Salvianolic acids are effective against several AS-related cellular components and molecular targets, but their safety remains to be assessed. Acute toxicity studies revealed a median lethal dose (LD50) of 2,356.33 mg/kg for DSS in mice, while rat studies demonstrated an excellent safety profile with no mortality or toxicity signs at 1,500 mg/kg (Gao et al., 2009). Subchronic toxicity assessments were used to further confirm the safety of DSS, which showed no mortality or histopathological changes in rats following 90 d of administration at doses up to 450 mg/kg (Gao et al., 2009). Injection-site sclerosis was seen only in the high-dose group after 2–3 weeks of continuous administration (normalized after 3–4 d of stopping the drug), according to a toxicity evaluation of DSS in Beagle dogs after 3 months of continuous infusion at doses of 17, 50, and 150 mg/kg/d (Li et al., 2009). Acute toxicity studies showed an LD50 of 1,161.2 mg/kg for SAA in mice without pathological abnormalities, while studies on Beagle dogs revealed an minimum lethal dose (MLD) of 682 mg/kg and maximum non-lethal dose (MNLD) of 455 mg/kg accompanied by hepatic and renal pathologies. A 4-week repeated-dose study identified dose-dependent toxicity at 300 mg/kg, which manifested as reduced hemoglobin level, decreased hematocrit, and hepatorenal impairment (Yang et al., 2022). Genotoxicity assessments through bacterial reverse mutation and mouse bone marrow micronucleus tests demonstrated no mutagenic effects or hematopoietic function alterations (Yang et al., 2022). Nephrotoxicity is a significant side-effect of the clinical use of cisplatin. Interestingly, DSS (60 mg/kg) protects against cisplatin-induced nephrotoxicity by activating Nrf2/HO-1 and blocking the NF-κB pathway (Yu et al., 2021). Magnesium lithospermate B as a magnesium salt derivative of SAB has shown nephroprotective effects by mitigating cisplatin-induced acute kidney injury through preservation of mitochondrial function (Shen et al., 2022). Furthermore, SAB (2 mg/kg) demonstrates cardioprotective synergy with arsenic trioxide (ATO) by alleviating ATO-induced cardiac dysfunction and myocardial damage (Wang et al., 2013).

Clinical evaluation in a phase I randomized double-blind placebo-controlled trial confirmed the excellent safety profile of SAB injection in healthy Chinese volunteers for single doses up to 300 mg and repeated doses of 250 mg for 5 d (Cheng et al., 2023). A single intravenous infusion of 100 or 200 mg of salvia polyphenols was tolerated well by the participants in a randomized open single-dose study of the compound in a healthy population. Salvianolic acid salt (85% SAB concentration) was evaluated for its impacts on platelets in patients with acute coronary syndrome (ACS); the results showed that it did not affect hemostasis in ACS patients and improved the antiplatelet effects of conventional antiplatelet medication (Liu et al., 2014). According to a different study, SAA also prevented platelet aggregation and activation in diabetic individuals but their diabetes-related comorbidities did not lower this inhibitory impact (Zhou et al., 2020).

The above information indicates that although the safety, tolerability, and effects of salvianolic acids on platelet augmentation have all been investigated partially, high-quality evidence is still required to support clinical usage of the drug as a novel antiplatelet treatment. A comprehensive literature review across PubMed, Web of Science, and Embase revealed that there were no existing studies on the reproductive toxicity of salvianolic acid, including fertility, embryofetal development, and perinatal effects. This gap likely stems from two factors: (1) historical research emphasis on pharmacodynamic mechanisms rather than systematic toxicity assessments; (2) the substantial time and financial investments required for ICH S5(R3)-compliant multigenerational reproductive toxicity studies. To address these, future research should implement a tiered approach of initial developmental toxicity screening using zebrafish embryo models, followed by extended one-generation reproductive toxicity studies in SD rats with detailed monitoring of F1 generation reproductive organ histopathology and sex hormone dynamics. Ultimately, large-scale multicenter clinical trials (such as phase IV pharmacovigilance studies) should establish human risk prediction models, thereby completing the safety profile of salvianolic acids and supporting its evidence-based clinical applications.

## 5 Drug interactions

Several studies have demonstrated the potential therapeutic roles of salvianolic acids in AS-related diseases. Compared to controls who received rosuvastatin alone, concurrent treatment with DSS (46 mg/kg) increased systemic exposure to rosuvastatin by more than twice and lowered plasma clearance to more than 57%. According to the current findings, CYPs and/or organic anion-transporting peptides may mediate these interactions (Wen and Xiong, 2011). The Cmax of DSS increased from 0.05 mg/mL to 0.11 mg/mL, while the area under the curve (AUC) value increased from 5.20 mg/mL·min to 18.47 mg/mL·min when combined with warfarin (Zhou et al., 2012). However, aspirin had no discernible impacts on the pharmacokinetic characteristics of DSS (Zhou et al., 2012). According to another investigation, DSS considerably increased the plasma levels of aspirin (Zhang et al., 2020). Thus, when consuming warfarin or aspirin along with herbal remedies and preparations containing DSS, it is necessary to monitor their plasma concentrations. Coadministration of DSS and irbesartan changed the metabolism of irbesartan by increasing its AUC_(0-t)_ (9,573 ± 441 vs. 16,157 ± 559 μg/L·h) and Cmax (821 ± 24 vs. 1,231 ± 44 μg/L), thereby prolonging t_1/2_ (13.39 ± 0.98 vs. 16.04 ± 1.21 h), decreasing clearance, improving the stability of *in vitro* metabolism, and lowering endogenous clearance (from 38.14 ± 10.24 μL/min/mg protein to 28.51 ± 9.06 μL/min/mg protein) (Li and Liu, 2024).

Regarding the drug interactions with DSS as a natural medication, strong cardioprotective impacts of DSS were observed when used in conjunction with salvinorin, which may be linked to the regulation of Bax, Bcl-2, and calpain I to exert antiapoptotic effects as well as activation of Nrf2 to boost the antioxidant effects (Li et al., 2012a). According to reports, the combination of SAB and astragaloside IV (AsIV) can effectively prevent and treat AS; this combination can positively affect lipid metabolism, antioxidative stress, and protection of the vascular endothelium. However, compared to the administration of single agents, the combination of SAB and AsIV had a more substantial antioxidant impact and an inhibitory effect on lipid deposition in the artery walls (Kong et al., 2022). Ligustrazine and SAB were observed to decrease the late apoptotic rate of rCMECs under high-shear flow conditions; however, their combination lowered the persistence of apoptosis rather than shielding cells from early death, inhibiting apoptosis synergistically (Li et al., 2008). By restoring the balanced production of proapoptotic and antiapoptotic proteins as well as the balanced redox state of cells through p38MAPK/NF-κB and PI3K/AKT/NF-κB, SAB and ginsenosides have been shown to produce concerted effects to counteract apoptosis (Yang et al., 2018). In the zebrafish vasculature, cotreatment with SAB and ferulic acid increased the expressions of specific VEGF receptors and ligands while promoting the proliferation and cell cycle progression of endothelial cells. The AUC and Cmax of SAB increased by 51.7% and 27.7%, respectively, when coupled with metoprolol (a common medication used to treat hypertension); when mixed with metoprolol acid, the AUC and Cmax of SAB decreased by 26.5% and 19.6%, respectively (Wan et al., 2010). Briefly, DSS and SAB produce synergistic effects with certain drugs and herbal monomers in treating AS, lipid-lowering, antiplatelet, anticoagulation, and antihypertensive disorders. Notably, the drug interactions of SAA with associated medications for AS therapy are rarely reported. Hence, further research efforts are necessary to determine whether the addition of salvianolic acids to traditional anti-atherosclerotic medications may lower or enhance the likelihood of their side-effects, such as hepatic impairment and elevated risk of bleeding.

## 6 Pharmacokinetic characteristics of salvianolic acids and their preparations

Salvianolic acids and their preparations have been proven to have potential therapeutic effects in AS-related disorders via numerous studies. Table 2 lists the pharmacokinetic properties of these preparations in various people and animals. Comparative pharmacokinetic analysis across species (rats, rabbits, beagles, and humans) have revealed significant interspecies variations in the absorption kinetics, metabolic pathways, and elimination profiles of salvianolic acids (Table 2). Beagles emerged as the most relevant preclinical model for oral formulation development, given that their gastrointestinal physiology (including pH environment and transit time) closely resembles that of humans. Conversely, rat-derived pharmacokinetic data require cautious clinical extrapolation owing to species-specific differences, such as significantly enhanced hepatic CYP450 metabolic activity and pronounced first-pass effects that may substantially underestimate systemic drug exposure. However, distribution metabolism studies have revealed that the primary metabolic reaction of DSS in rats is the formation of methylated DSS, with 4-hydroxy-3-methoxyphenyllactic acid as the main metabolite (Wang et al., 2015b). SAA has two metabolic pathways in rats, including methylation and glucuronidation, which produce five metabolites (SAA-monoglucuronide, monomethyl-SAA-monoglucuronide, monomethyl-SAA, dimethyl-SAA, and dimethyl-SAA-monoglucuronide) (Shen et al., 2009). Similarly, the primary metabolic pathway of SAB in rats is methylation that produces nine metabolites, including methylated metabolites of SAB and lithospermic acid (LSA), as well as decarboxylation and methylation metabolites of LSA, salvianolic acid S (SAS), and dehydrated SAS (Qi et al., 2013).

**TABLE 2:** Pharmacokinetic properties of salvianolic acids and their preparations**.**

Salvianolic acids	Species	Dose	Pharmacokinetic parameters	Reference
DSS	Rats	Danshen injection (amount of DSS is 0.701 g/L)	T_1/2_ = 0.42 ± 0.03 min	Chang et al. (2010)
			AUC_0–t_ = 2.09 ± 0.42 μg·h/mL	
			AUC_0–∞_ = 2.12 ± 0.43 μg·h/mL	
			MRT = 0.31 ± 0.04 h	
			CL = 3.42 ± 0.72 L/h/kg	
			V_d_ = 2.09 ± 0.54 L/kg	
		Danhong injection (amount of DSS is 3.24 mg/kg)	T_1/2_ = 4.48 ± 3.98 h	Nijat et al. (2022)
			T_max_ = 0.25 ± 0.00 h	
			C_max_ = 0.77 ± 0.23 μg/mL	
			AUC_0–12_ = 0.99 ± 0.43 μg·h/mL	
			MRT = 0.23 ± 0.09 h	
		1.3 g/kg of Qiliqiangxin capsule (amount of DSS is 1.770 mg/g)	T_1/2_ = 4.28 ± 1.42 h	Zhang et al. (2018)
			T_max_ = 1.50 ± 0.24 h	
			C_max_ = 0.13 ± 0.02 μg/mL	
			AUC_0–t_ = 0.37 ± 0.04 μg·h/mL	
			AUC_0–∞_ = 0.42 ± 0.03 μg·h/mL	
	Rabbit	0.4 g/mL of DSS	T_1/2_ = 0.14 ± 0.07 h	Li et al. (2007)
			AUC = 36.05 ± 28.53 μg·h/mL	
			CL = 0.32 ± 0.24 L/h/kg	
	Beagle dogs	60 mg/kg of DSS (oral)	C_max_ = 7.92 ± 1.77 μg/mL	Jiang et al. (2017)
			T_1/2_ = 1.84 ± 0.29 h	
			T_max_ = 2.83 ± 0.41 h	
			MRT = 4.23 ± 0.20 h	
			AUC_0–t_ = 22.21 ± 2.48 μg·h/mL	
			AUC_0–∞_ = 22.73 ± 2.57 μg·h/mL	
		15 mg/kg of DSS (i.v.)	C_0_ = 79.15 ± 18.92 μg/mL	
			T_1/2_ = 1.64 ± 0.39 h	
			MRT = 0.73 ± 0.05 h	
			CL = 8.67 ± 1.83 L/h	
			V_d_ = 6.36 ± 1.48 L	
			AUC_0–t_ = 17.75 ± 3.03 μg·h/mL	
			AUC_0–∞_ = 17.79 ± 3.04 μg·h/mL	
	Humans	Compound danshen dripping pills	C_max_ = 134.1 ± 48.4 μg/mL	Li et al. (2017)
			T_max_ = 0.67 ± 0.25 h	
			T_1/2_ = 1.68 ± 0.56 h	
			MRT = 1.87 ± 0.29 h	
			AUC_0–t_ = 0.22 ± 0.07 μg·h/mL	
			AUC_0–∞_ = 0.23 ± 0.07 μg·h/mL	
SAA	Rats	Danshen injection (amount of SAA is 0.0121 g/L)	T_1/2_ = 0.96 ± 0.20 h	Chang et al. (2010)
			AUC_0–t_ = 0.38 ± 0.08 μg·h/mL	
			AUC_0–∞_ = 0.39 ± 0.09 μg·h/mL	
			MRT = 0.50 ± 0.12 h	
			CL = 0.32 ± 0.07 L/h/kg	
			V_d_ = 0.45 ± 0.16 L/kg	
		Danhong injection (amount of SAA is 0.9 mg/kg)	T_1/2_ = 6.47 ± 3.60 h	Nijat et al. (2022)
			T_max_ = 0.25 ± 0.00 h	
			C_max_ = 0.05 ± 0.02 μg/mL	
			AUC_0–12_ = 0.12 ± 0.05 μg·h/mL	
			MRT = 2.95 ± 1.04 h	
		1.3 g/kg of Qiliqiangxin capsule (amount of SAA is 0.772 mg/g)	T_1/2_ = 17.88 ± 3.88 h	Zhang et al. (2018)
			T_max_ = 1.00 ± 0.00 h	
			C_max_ = 0.01 ± 0.00 μg/mL	
			AUC_0–t_ = 0.01 ± 0.00 μg·h/mL	
			AUC_0–∞_ = 0.02 ± 0.01 μg·h/mL	
	Beagle dogs	10 mg/kg of SAA (oral)	AUC_0–t_ = 0.08 ± 0.03 μg·h/mL	Sun et al. (2013)
			C_max_ = 0.03 ± 0.01 μg/mL	
			T_max_ = 1.73 ± 2.85 h	
			T_1/2_ = 1.20 ± 0.44 h	
			MRT_0–t_ = 4.87 ± 2.09 h	
			F = 1.84 ± 0.74%	
		20 mg/kg of SAA (i.v.)	AUC_0–t_ = 0.13 ± 0.10 μg·h/mL	
			C_max_ = 0.04 ± 0.02 μg/mL	
			T_max_ = 0.68 ± 0.82 h	
			T_1/2_ = 3.00 ± 0.79 h	
			MRT_0–t_ = 5.85 ± 4.69 h	
			F = 1.47 ± 1.08%	
	Humans	300 mg of SAA	C_max_ = 14.31 ng/mL	Chen et al. (2022c)
			AUC_0–t_ = 14.56 μg·h/mL	
			AUC_0–∞_ = 14.66 μg·h/mL	
			T_1/2_ = 2.92 h	
			V_d_ = 87.7 L	
			CL = 21.2 L/h	
SAB	Rats	Danshen injection (amount of SAB is 0.163 g/L)	T_1/2_ = 0.75 ± 0.08 h	Chang et al. (2010)
			AUC_0–t_ = 0.46 ± 0.09 μg·h/mL	
			AUC_0–∞_ = 0.56 ± 0.11 μg·h/mL	
			MRT = 0.75 ± 0.06 h	
			CL = 3.00 ± 0.66 L/h/kg	
			V_d_ = 3.27 ± 0.99 L/kg	
		4.8 g/kg/d of Yindanxinnaotong capsule	C_max_ = 0.03 ± 0.02 μg/mL	Gong et al. (2018)
			T_max_ = 0.75 ± 0.18 h	
			AUC_0–t_ = 0.12 ± 0.06 μg·h/mL	
			AUC_0–∞_ = 0.14 ± 0.06 μg·h/mL	
			T_1/2_ = 12.17 ± 3.33 h	
			MRT = 9.46 ± 2.37 h	
		Danhong injection (amount of SAB is 1.17 mg/kg)	T_1/2_ = 13.57 ± 17.65 h	Nijat et al. (2022)
			T_max_ = 0.25 ± 0.00 h	
			C_max_ = 0.11 ± 0.02 μg/mL	
			AUC_0–12_ = 0.17 ± 0.06 μg·h/mL	
			MRT = 0.68 ± 0.29 h	
		1.3 g/kg of Qiliqiangxin capsule (amount of SAB is 3.987 mg/g)	T_1/2_ = 25.88 ± 1.44 h	Zhang et al. (2018)
			T_max_ = 1.00 ± 0.00 h	
			C_max_ = 0.01 ± 0.00 μg/mL	
			AUC_0–t_ = 0.07 ± 0.01 μg·h/mL	
			AUC_0–∞_ = 0.20 ± 0.05 μg·h/mL	
	Rabbit	5.25 mg/kg of SAB (i.v.)	T_1/2_ = 3.07 ± 1.86 h	Ma and Wang (2007)
			MRT = 2.89 ± 2.36 h	
			V_d_ = 4.61 ± 4.20 L/kg	
			CL = 0.94 ± 0.27 L/h/kg	
			AUC_0–n_ = 5.48 ± 1.67 μg·h/mL	
			AUC_0–∞_ = 5.96 ± 1.64 μg·h/mL	
	Humans	150 mg/kg of SAB	T_max_ = 0.97 ± 0.09 h	Cheng et al. (2023)
			T_1/2_ = 1.45 ± 0.50 h	
			C_max_ = 8.65 ± 2.58 μg/mL	
			AUC_0–t_ = 10.24 ± 3.99 μg·h/mL	
			AUC_0–∞_ = 10.40 ± 4.03 μg·h/mL CL = 16.27 ± 5.63 L/h	

Salvianolic acids have limited oral bioavailability, similar to many natural compounds. Among these, DSS has 13.72% absolute bioavailability and brief duration of body circulation (Meng et al., 2019). According to preclinical research, the absolute oral bioavailabilities of SAA are roughly 0.6% and 1.84% in rats and beagles, respectively (Sun et al., 2013). This is mainly because the substance is found in an ionic state in the digestive system and has low bioavailability owing to its difficulty passing through the intestinal biomembrane (Chen et al., 2022c). Furthermore, because SAB has poor permeability and stability, its oral bioavailability in rats is only around 2.3% (Wu et al., 2006). The above issues can be successfully resolved with the advancement of pharmaceutical research and creation of novel formulations. Tanshinol borneol ester was loaded into nanostructured lipid carriers modified with polyethylene glycol and administered intravenously; the results of this effort were a significant increase in the area under the blood concentration vs. time curve, longer plasma retention time, and lower clearance. Hence, this formulation is the recommended option for the sustained release of DSS *in vivo* and may be among the most promising therapies for managing cardiovascular disorders (Yuan et al., 2018). To increase oral bioavailability, a solid self-microemulsifying drug delivery system was developed in another study and loaded with many active metabolites of *S. miltiorrhiza* Bunge as a novel approach to oral medication delivery (Bi et al., 2016).

Because of their structural characteristics, salvianolic acids have low absorption, hampering their clinical applicability even as promising natural substances in AS-related diseases. Novel advancements in materials sciences and related pharmaceutical sciences have made it feasible to load drug using a range of cutting-edge materials, significantly boosting the bioavailability and precisely delivering to the drug targets via various pathways. However, several critical aspects of salvianolic acids require further investigations and optimization, including development of pharmaceutical formulations to enhance stability and bioavailability, comprehensive evaluations of efficacy and safety in AS-related models, and validation of stability-enhancing technologies to facilitate clinical translation. These advancements are essential for promoting the clinical application and therapeutic potential of salvianolic acids.

## 7 Conclusion and future directions

Numerous research studies have demonstrated the positive pharmacological and therapeutic effects of salvianolic acids on AS. Salvianolic acids can influence the actions of several cellular components, such as endothelial cells, smooth muscle cells, macrophages, EPCs, and other immune cells, which are linked to the development of AS and can slow its progression. The therapeutic benefits of SAB, SAA, and DSS for AS include multitarget and multipathway effects that are strongly linked to the regulation of oxidative stress, inflammation, autophagy, apoptosis, platelet aggregation, thrombosis, vascular tone, and other processes. However, the current study entails specific practical issues that prevent salvianolic acids from being used in clinical settings in the future.

### 7.1 Clarifying direct action targets

Published studies do not thoroughly examine the deeper mechanisms and particular action connections of the anti-atherosclerotic properties of salvianolic acids. Salvianolic acids have tremendous potential in the treatment of AS, as demonstrated by their ability to influence multilayer signaling cascades. In light of the available literature, we analyzed the anti-atherosclerotic cellular components and related mechanisms of SAA, SAB, and DSS. However, previous studies have not examined the precise active metabolic components; instead, these studies concentrated on a single cellular element or method of inhibiting autophagy, apoptosis, and inflammation. For instance, the degree of lipid metabolism in macrophages plays a significant role in controlling AS. To date, most studies have only examined whether salvianolic acids can help control the levels of lipid metabolism rather than the upstream mechanisms of this effect. To systematically explore the direct targets of salvianolic acids, the following integrated strategies are proposed. First, multiomics integration can be employed to construct a multitarget regulatory network complemented by molecular docking and molecular dynamics simulations to predict the binding modes between salvianolic acids and their targets, thereby prioritizing candidates for experimental validation. Second, two distinct types of bioprobes can be developed: (1) bioorthogonal probes derived from the core structures of salvianolic acids to enable target visualization and enrichment via click chemistry; (2) activity-based probes of salvianolic acid analogs that can covalently label target proteins for precise identification through mass spectrometry. Additionally, cellular thermal shift analysis may be used to assess changes in the thermal stability of the target protein upon salvianolic acid binding to provide orthogonal validation. This synergistic approach combining multiomics analysis, computational modeling, and chemical biology techniques offers a reliable experimental basis for comprehensively elucidating the direct molecular targets of salvianolic acids.

### 7.2 Strengthening clinical evidence

The clinical evidence base for salvianolic acids in AS management remains limited and has several methodological constraints: (1) current AS-related trials predominantly consist of single-center small-scale exploratory studies lacking rigorous randomization, blinding, or placebo controls; (2) unoptimized dosing regimens due to insufficient dose-response studies have led to inconsistent efficacy evaluation criteria; (3) while randomized controlled trials (RCTs) of salvianolic acid injection have demonstrated efficacy in cerebral perfusion disorders, these neurological-focused findings cannot be directly extrapolated to AS management; (4) existing safety data are primarily derived from short-term assessments in healthy populations, with inadequate representation of high-risk subgroups (such as diabetic patients or those with vulnerable plaques), thereby limiting the clinical generalizability of the current findings.

Future research efforts should therefore adopt comprehensive strategies to strengthen the evidence chain: (1) conduct multicenter large-scale randomized double-blind placebo-controlled phase II/III trials to evaluate the efficacies of salvianolic acids, both as monotherapy and in combination with standard care (such as statins), on the hard endpoints of AS (such as major adverse cardiovascular events and plaque regression); (2) implement population pharmacokinetic and pharmacodynamic (PPK-PD) modeling to optimize personalized dosing strategies; (3) incorporate real-world evidence (such as electronic health records) to assess the efficacy and safety in complex patient populations, thereby addressing the external validity limitations of RCT findings.

### 7.3 Clarifying the effectiveness and safety of drug interactions

Emerging evidence indicates the synergistic potential between salvianolic acids and conventional anti-atherosclerotic therapies, although mechanistic elucidation and clinical translation demand rigorous validation. While the current investigations predominantly address pharmacokinetic parameters, three critical knowledge gaps require systematic exploration. The first gap is statin synergism, where the proposed complementary mechanisms between salvianolic-acid-mediated ABCA1/PPAR-γ/LXRα activation and statin-induced HMG-CoA reductase inhibition necessitate quantitative validation. Here, the essential research efforts should prioritize (a) systematic characterization of CYP3A4 inhibitory potency of salvianolic acids and the dose-dependent effects on statin metabolism as well as (b) multicenter RCTs evaluating the efficacy of combination therapy in plaque stabilization and cardiovascular mortality reduction, with standardized monitoring of the hepatic transaminases and creatinine kinase levels. The second gap involves anticoagulant interactions, where the potential pharmacodynamic interactions require rigorous investigation through (a) warfarin metabolism inhibition and plasma protein binding competition, which may elevate the AUC and INR values as well as (b) synergistic COX-1 inhibition with aspirin to potentially enhance the antiplatelet effects while increasing the risk of gastrointestinal bleeding. Development of pharmacodynamic models and patient-specific monitoring protocols (INR, fecal occult blood) based on individual risk factors (age, ulcer history) is also essential. The third gap entails multicomponent synergism, where systematic analyses of the effects of different isomers of salvianolic acids are needed on CYP450 isoforms, transporters, and signaling pathways through *in vitro* screening and AS animal models to elucidate the multicomponent synergistic mechanisms and optimize clinical combination strategies.

### 7.4 Pharmacological mechanisms and comparative advantages

Comparative analyses of some natural anti-atherosclerotic agents reveal distinct yet complementary mechanisms. (1) Resveratrol (RSV): This agent primarily targets endothelial regulation through SIRT1/AMPK/eNOS activation and TGF/ERK pathway inhibition to improve vasodilation and suppress smooth muscle proliferation (Guo et al., 2022; Parsamanesh et al., 2021). (2) Curcumin: This agent focuses on NLRP3 inflammasome-mediated inflammation to modulate macrophage polarization (inhibiting iron-induced M1 phenotype) and platelet activation (reducing P-selectin expression), albeit with limited endothelial repair capacity (Benameur et al., 2023; Hussain et al., 2022; Momtazi-Borojeni et al., 2019). (3) Quercetin: This is a dietary flavonoid requiring intestinal microbial metabolism for activation that exerts antioxidant (LDL reduction) and anti-inflammatory effects while indirectly improving lipid metabolism through gut microbiota modulation. However, the clinical outcomes remain inconsistent owing to bioavailability limitations and individual metabolic variations (Papakyriakopoulou et al., 2022; Terao, 2023). (4) Salvianolic acids demonstrate superior multitarget efficacy through YAP/TAZ/JNK-NLRP3 axis inhibition to block endothelial inflammation and pyroptosis; SIRT1-mediated autophagy activation for oxidative stress clearance; MEK/ERK pathway modulation to inhibit endothelial apoptosis and promote progenitor cell differentiation; and comprehensive regulation of smooth muscle migration, macrophage lipid metabolism, and platelet activation. Unlike the endothelial focus of RSV, anti-inflammatory specialization of curcumin, or microbiota-dependent effects of quercetin, salvianolic acids offer a systematic therapeutic approach encompassing endothelial homeostasis, plaque stabilization, and vascular regeneration. Future research efforts could thus employ systems pharmacology procedures to explore natural product synergies (such as salvianolic acid and curcumin nanoformulations) and utilize advanced models (organoid arrays, patient-derived plaque systems) to quantitatively assess target engagement and pathway modulation efficiency to optimize clinical applications.

In conclusion, the primary water-soluble metabolites of *S. miltiorrhiza* Bunge show sound vasoprotective effects in the treatment of AS and can be used as candidate drugs for the prevention and treatment of AS owing to its intricate pathological mechanisms and complex interactions between different cellular components. However, there remain certain disadvantages and shortcomings in the practical applications of salvianolic acids because of their low stability, significant individual variances, lack of clinical trial data, and numerous constraints. Future advancements in the clinical applications and development of salvianolic acids for the treatment of AS-related diseases will require more investigations of the interactions, high-quality clinical trials, optimization of drug formulations and dosing regimens, as well as integration of research and development of novel formulations, modern technological tools like artificial intelligence and computer science, and in-depth studies in molecular biology and pharmacology. These efforts are expected to yield valuable drug templates for advanced clinical applications.
